# Optical Access to Dark Excitons Via Spin–Phonon Fano Interference

**DOI:** 10.1002/advs.78207

**Published:** 2026-10-14

**Authors:** Janina J. Schindler, Katja Barthelmi, Mikhail M. Glazov, Carlo Sgroi, Sven Scholz, Arne Ludwig, Andreas D. Wieck, Jörg Debus

**Affiliations:** ^1^ Department of Physics TU Dortmund University Dortmund Germany; ^2^ Ioffe Institute St. Petersburg Russia; ^3^ Experimental Physics VI, Faculty of Physics and Astronomy Ruhr University Bochum Bochum Germany; ^4^ Dortmund Center for Advanced Exploration of Dynamics Across Limits Using Spectroscopy (DAEDALUS) TU Dortmund University Dortmund Germany

**Keywords:** acoustic phonons, brillouin scattering, exciton, fano resonance, optical probing, quantum dot, resonant scattering, semiconductor nanostructures, spin dynamics

## Abstract

Spin‐dark excitons in semiconductor nanostructures are inherently optically silent: angular‐momentum selection rules forbid their radiative recombination, granting long lifetimes attractive for quantum information storage and excitonic condensation, but placing their energy structure and bright–dark coupling beyond standard optical spectroscopy. Established access routes rely on symmetry‐lowering perturbations, dedicated single‐emitter device fabrication, or near‐field methods, each of which perturbs the states under study or excludes ensembles. Here, we show that a Fano‐type quantum interference between two acoustic–phonon scattering pathways, consisting of a resonant spin‐flip process coupling bright and dark excitons and a non‐resonant inelastic process sharing the same acoustic phonon mode, opens an optical window onto the dark exciton in unperturbed ensembles of (In,Ga)As/GaAs quantum dots. The interference manifests as antiresonance and bipolar spectral profiles whose shape and position encode the bright–dark exciton coupling, are tunable through magnetic‐field orientation, optical pumping intensity, and temperature, and persist to about 40 K, with a characteristic thermal scale of about 5 meV. The same lineshape analysis reveals a sign reversal of the exciton exchange energy at excited quantum‐dot shells. Relying only on exchange‐split exciton states and acoustic phonons, the mechanism should transfer to two‐dimensional transition metal dichalcogenides and perovskite nanocrystals, where dark excitons play a central role.

## Graphical abstract

Dark excitons in quantum dots neither absorb nor emit light and stay hidden from optical spectroscopy. Quantum interference now reveals them: light scattered by acoustic phonons interferes with a phonon‐assisted spin flip between bright and dark exciton, carving a Fano dip into the spectrum whose position gives the bright–dark splitting. Magnetic field, laser power and temperature tune the interference.

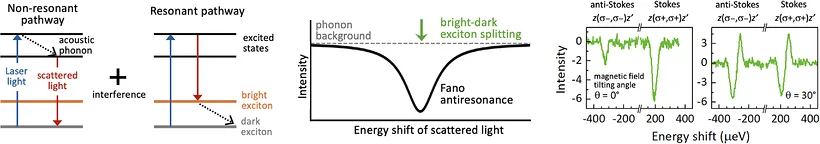

## 1 Introduction

Optically dark electronic states, those decoupled from the radiation field by symmetry, play a central role across nanoscience, from long‐lived spin qubits in semiconductor quantum dots (QDs) to spin‐forbidden excitons that dominate the low‐temperature photophysics of two‐dimensional (2D) transition metal dichalcogenides [1, 2, 3] and lead‐halide perovskite nanocrystals. The very property that makes such states attractive for quantum information storage and for proposed excitonic Bose–Einstein condensates [4, 5], namely the suppression of radiative decay, also places them beyond the reach of conventional optical spectroscopy [6]. Knowing the energy hierarchy of bright and dark states, the exchange coupling between them, and their environment‐induced relaxation pathways is nevertheless essential for understanding radiative properties, spin dynamics, and valley physics in modern nanostructures [7, 8].

In semiconductor QDs the relevant dark states are spin‐dark excitons, electron–hole pairs whose parallel carrier spins forbid radiative recombination through the dipole selection rules. Their lifetimes exceed those of their bright counterparts by up to six orders of magnitude, typically below 1 ns for the bright exciton [9] vs. about 1 ms for the dark exciton [10, 11], which is precisely why dark excitons are interesting and why they are hard to see. Existing strategies to probe them rely on symmetry‐lowering perturbations: strong in‐plane magnetic fields mix bright and dark spin states in QDs as well as in 2D transition metal dichalcogenides and perovskites [12, 13, 14, 15, 16, 17, 18], biexcitonic spin‐triplet cascades provide indirect access [19], QD microlenses enhance photon extraction [20], and tip‐enhanced nano‐optical methods induce dark exciton emission at room temperature [21]. Each of these approaches either requires dedicated device fabrication, operates only on single emitters, or introduces external perturbations that alter the states under study. A spectroscopic method that probes the dark exciton within an unperturbed ensemble and yields quantitative information on the bright–dark coupling has remained elusive.

In this work, we close this gap by exploiting a quantum‐interference channel that arises when an acoustic phonon mediates two competing scattering pathways simultaneously: one that resonantly flips the exciton spin between the bright and dark manifolds, and one that scatters inelastically without altering the spin. The interference between them imprints a Fano‐type signature on the optical spectrum from which the bright–dark exciton splitting can be extracted. Two features distinguish this mechanism from previously reported Fano resonances in semiconductor nanostructures, where the discrete state is typically a charged‐exciton or polaritonic transition coupled to a photonic continuum [22, 23, 24]. First, the discrete state here is the spin‐forbidden bright–dark transition itself, so the lineshape directly encodes the bright–dark coupling rather than reporting it indirectly through optically active states. Second, the occupation of the dark exciton is not a passive consequence of the selection rules but an active prerequisite of the mechanism: under continuous resonant excitation a steady‐state dark population is maintained by the pumping cycle and serves as the initial state for the Fano process. Together, these features turn the dark exciton from an optically silent state into the central element of an interferometric optical probe. We demonstrate the resulting spectroscopy in (In,Ga)As/GaAs QDs, extract its quantitative parameters as functions of magnetic‐field orientation, optical pumping intensity, and temperature up to 40 K, and use it to uncover a sign reversal of the exciton exchange energy at excited shells: a finding that opens the same approach to two‐dimensional transition metal dichalcogenides and perovskite nanocrystals, where dark‐exciton fine structure remains contested.

## 2 Results

### 2.1 Exciton Fine Structure and Spin–Phonon Fano Model

We label the four QD exciton states by their total angular–momentum projection: the bright states $\left|+1\right\rangle =|-\frac{1}{2},+\frac{3}{2}\rangle$ and $\left|-1\right\rangle =|+\frac{1}{2},-\frac{3}{2}\rangle$, created by $\sigma^{+}$ and $\sigma^{-}$ light, respectively, and the dark states $\left|+2\right\rangle =|+\frac{1}{2},+\frac{3}{2}\rangle$ and $\left|-2\right\rangle =|-\frac{1}{2},-\frac{3}{2}\rangle$, in which the electron (el) spin is parallel to the heavy‐hole (hh) spin. In the notation $|S_{z}^{\mathrm{el}},J_{z}^{\mathrm{hh}}\rangle$ the first entry is the projection of the electron spin, $S_{z}^{\mathrm{el}}=\pm \frac{1}{2}$, and the second that of the heavy‐hole angular momentum, $J_{z}^{\mathrm{hh}}=\pm \frac{3}{2}$, onto the QD growth axis z; light holes are split off by strain and confinement and are not considered. Recombination of the dark states requires a transfer of angular momentum ±2 and is therefore electric–dipole forbidden; neither the dark exciton energy nor the electron spin‐flip mechanism is directly accessible by optical means.

The longitudinal magnetic‐field‐dependent energies of the bright (b), $|\Psi_{b}\rangle =\left|\pm 1\right\rangle$, and dark (d), $|\Psi_{d}\rangle =\left|\pm 2\right\rangle$, exciton (X) states are calculated by $E_{X,\pm 1}=\frac{1}{2}\left(\delta_{0}\pm \sqrt{\delta_{b}^{2}+E_{Z,1}^{2}}\right)$ and $E_{X,\pm 2}=\frac{1}{2}\left(-\delta_{0}\mp \sqrt{\delta_{d}^{2}+E_{Z,2}^{2}}\right)$ [25, 26], where the band gap and exciton binding energy are incorporated into the energy origin, taken at the center of the exciton quadruplet. The exchange splitting $\delta_{0}$ between the bright and dark exciton is shell‐dependent and, as demonstrated experimentally below, takes the value $\delta_{0}=-75\;\mu \mathrm{eV}$ for the *p*‐shell. The negative sign indicates that the bright exciton is lowest in energy, in contrast to the conventional *s*‐shell ordering where the dark exciton is lowest. The zero‐field splitting of the bright (dark) exciton states is estimated to $\delta_{b}=4\;\mu \mathrm{eV}$ (similarly, the dark state splitting $\delta_{d}=1\;\mu \mathrm{eV}$) [11, 27]. The Zeeman (Z) energy is given by $E_{Z,m}=\mu_{B}\left[g_{z}^{\mathrm{hh}}-{\left(-1\right)}^{m}g_{z}^{\mathrm{el}}\right]B$ with the Bohr magneton $\mu_{B}$, m being the absolute value of the exciton angular momentum component (m=1 or 2 for the bright and dark excitons, respectively), and the strength B of the magnetic field which is applied along the QD growth axis z [26, 28]. The longitudinal g‐factors $g_{z}$ of the electron and heavy hole are −0.55 and −0.45, respectively [27, 29]. Since |±1⟩ and |±2⟩ share the same heavy‐hole state and differ only in the electron spin, one has, up to corrections of order $\delta_{b}^{2}/E_{Z,1}$ and $\delta_{d}^{2}/E_{Z,2}$, $\Delta E_{\mathrm{bd}\pm }=\delta_{0}\mp g_{z}^{\mathrm{el}}\mu_{B}B$; the hole g‐factor drops out of the bright–dark splitting. The magnetic‐field dependence of the exciton spin fine structure is presented in Figure 1a. Due to the nonzero exchange constants, the energies evolve asymmetrically with respect to $E_{X}=0$. This asymmetry also manifests itself in the transition from either |+1⟩ to |+2⟩ (initialized by $\sigma^{+}$ polarized light) or |−1⟩ to |−2⟩ ($\sigma^{-}$ initialization) which is realized by the electron spin‐flip. The respective energy differences $\Delta E_{\mathrm{bd}+}=E_{X,+1}-E_{X,+2}$ and $\Delta E_{\mathrm{bd}-}=E_{X,-1}-E_{X,-2}$ are governed by $\delta_{0}$ and the electron Zeeman splitting; they are depicted in Figure 1b. At B=8 T, they amount to about 180 and −330μeV, respectively.

**FIGURE 1:**
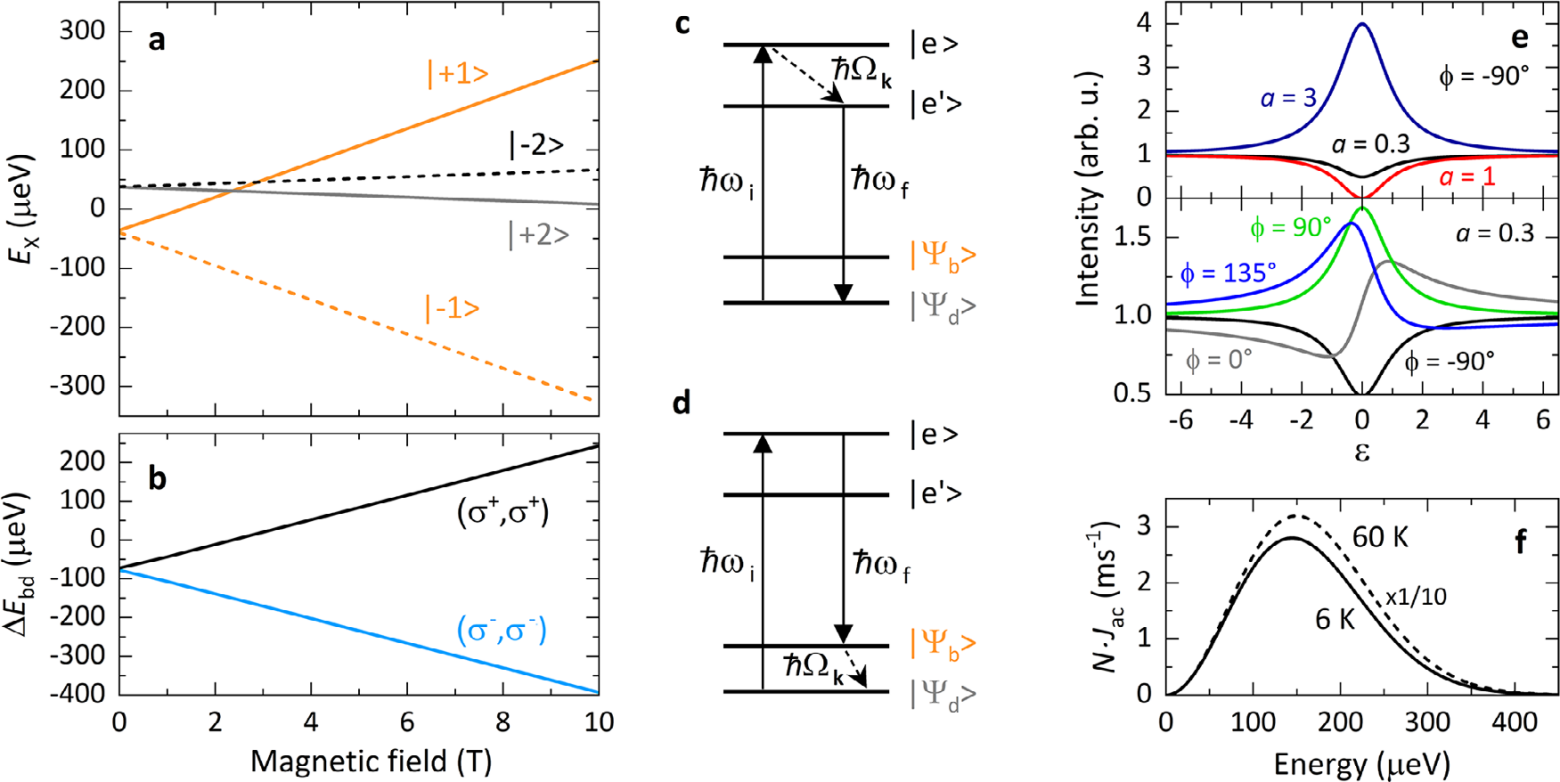
Theoretical details on the exciton energies, spin–phonon Fano resonance, Fano profile, and phonon spectral density. (a) Bright and dark exciton energies $E_{X,\pm 1}$ and $E_{X,\pm 2}$ calculated as a function of an external magnetic field in Faraday geometry for the *p*‐shell; the exchange energy $\delta_{0}=-75\;\mu \mathrm{eV}$ is taken from the experiment (Figure 2d). Note that the sign and magnitude of $\delta_{0}$ are shell‐dependent (Figure 2e). (b) Magnetic‐field‐dependent energy differences involving the |+1⟩→|+2⟩ and |−1⟩→|−2⟩ transitions given in $\sigma^{+}$ and $\sigma^{-}$ circular copolarizations, respectively. Scheme of the (c) non‐resonant and (d) resonant Fano process pathway. (e) Fano profiles calculated for different parameters a and ϕ after Equation (1). (f) Weighted spectral density of the transverse acoustic phonons in InGaAs QDs evaluated for 6 and 60 K.

The zero‐phonon optical recombination of the dark exciton becomes possible due to an admixture of optically active and dark exciton states. Admixtures may be established by reducing the symmetry of the system via, e.g., an oblique magnetic field yielding mixed electron or hole spin states [12, 13, 14, 15, 16, 17, 18]. In the absence of such external impacts reducing the symmetry, we reveal that a novel Fano resonance provides optical access to the spin‐dark exciton: it is based on the quantum interference of an acoustic phonon involved in both a non‐resonant spin‐conserving scattering as well as a resonant spin‐flip scattering process between the bright $|\Psi_{b}\rangle$ and dark exciton $|\Psi_{d}\rangle$. Our experiments clearly show resonant light scattering with an energy shift that matches the bright–dark exciton splitting $\Delta E_{\text{bd}}$. This observation calls for a scattering model in which the exciton states serve as both initial and final states of the process. Under continuous resonant excitation, bright excitons are steadily created in the *p*‐shell and a fraction undergoes spin‐flip scattering into the dark state. In steady state, the dark occupation is set by the balance between this spin‐flip channel and the spin‐conserving orbital relaxation that drains both spin states into the *s*‐shell, so that a stationary dark population is maintained for as long as the excitation is applied. Its magnitude does not enter the lineshape: both interfering pathways start from the same dark exciton, so within the linear‐response, unsaturated treatment used below the dark population appears only as a common prefactor of the scattered intensity and cancels from shape parameters, as shown in the following. What the Fano lineshape requires is therefore that the dark state be populated.

For illustration we consider the Stokes process and take the dark exciton $|\Psi_{d}\rangle$ as the initial state. The non‐resonant process (Figure 1c) is a kind of Brillouin–Mandelstam scattering where (i) an incident circularly polarized photon with energy $\hbar \omega_{i}$ transforms the exciton into an excited state |e⟩ [30], (ii) an acoustic phonon with energy $\hbar \Omega_{k}$ is emitted and the system goes to the excited state $|e^{\prime }\rangle$, and (iii) finally a photon is emitted, whose energy is given by $\hbar \omega_{f}=\hbar \omega_{i}\pm \hbar \Omega_{k}$ (minus sign corresponds to a Stokes process) and helicity is the same as that of the incident photon. The matrix element describing this non‐resonant process reads $M_{k}^{\mathrm{nr}}=M_{+2e^{\prime }}V_{e^{\prime }e}^{k}M_{e+2}{\left[\left(\hbar \omega_{i}-E_{e}+i\Gamma_{e}\right)\left(\hbar \omega_{i}-E_{e^{\prime }}-\hbar \Omega_{k}+i\Gamma_{e^{\prime }}\right)\right]}^{-1}$. Here, $E_{e}$ and $E_{e^{\prime }}$ are the energies of the excited states (measured from the dark exciton energy), $\Gamma_{e}$ and $\Gamma_{e^{\prime }}$ are their broadenings as well as $V_{e^{\prime }e}^{k}$ is the matrix element of the phonon‐induced scattering between the excited states. As the matrix element weakly depends on the incident photon energy and on the scattering energy shift given by the phonon dispersion $\hbar \Omega_{k}$, such a process is indeed non‐resonant.

The resonant process occurs via an exciton–phonon interaction yielding a change in the exciton spin state; in the case of Stokes scattering the exciton goes finally from |+1⟩ into the |+2⟩ state, as shown in Figure 1d. This process can be considered as initially a virtual exciton transition from the |+2⟩ to the excited state |e⟩ due to absorption of a circularly polarized photon followed by the emission of a co‐polarized photon yielding the transition to the |+1⟩ bright state. Eventually, a phonon is emitted, and the exciton is converted to a dark state. The matrix element of this process reads $M_{k}^{r}=V_{+2,+1}^{k}M_{+1e}M_{e+2}{\left[\left(\hbar \omega_{i}-E_{e}+i\Gamma_{e}\right)\left(\hbar \omega_{i}-\hbar \omega_{f}-\Delta E_{\mathrm{bd}+}+i\Gamma_{b}\right)\right]}^{-1}$. This process is characterized by a resonant denominator $\hbar \omega_{i}-\hbar \omega_{f}-\Delta E_{\mathrm{bd}+}+i\Gamma_{b}$ that vanishes as soon as the Raman shift equals the bright–dark splitting $\Delta E_{\mathrm{bd}+}$. Both the non‐resonant and resonant pathways interfere with each other, as they share the same initial (dark exciton and $\sigma^{\pm }$ photon) and final state ($\sigma^{\pm }$ photon and phonon k). Their diagrammatic representations are provided in the Supporting Information.

The intensity of the scattered light takes the form $I\propto \frac{2\pi }{\hbar }\sum_{k}{\left|M_{k}^{r}+M_{k}^{\mathrm{nr}}\right|}^{2}\delta \left(\hbar \omega_{i}-\hbar \omega_{f}-\hbar \Omega_{k}\right)$. Assuming a double‐resonant regime in which the incident photon is resonant with the intermediate state, $\left|\hbar \omega_{i}-E_{e}\right|\ll \Gamma_{e}$, and the scattered photon with the state $|e^{\prime }\rangle$, $\left|\hbar \omega_{f}-E_{e^{\prime }}\right|\ll \Gamma_{e^{\prime }}$, and matrix elements $V^{k}$ independent of the direction of k, we obtain the expression for the scattered light intensity in the form

(1) $$I\propto R\left(\omega_{i},\omega_{f}\right)\times \left[1+\frac{a^{2}+2a\left(\varepsilon \cos\phi +\sin\phi \right)}{\varepsilon^{2}+1}\right].$$

It describes an asymmetric Fano‐like line shape related to the interference between the resonant and non‐resonant scattering processes. Here, the reduced energy is $\varepsilon =\left(\hbar \omega_{i}-\hbar \omega_{f}-\Delta E_{\text{bd}+}\right)/\Gamma_{b}$, and $R\left(\omega_{i},\omega_{f}\right)$ is a smooth background function of the incident and scattered photon frequencies. $\Gamma_{b}$ is the same damping that appears in the resonant denominator above, written $\Gamma_{↑}$ in the Supporting Information. The analogous expressions for the |−1⟩→|−2⟩ channel follow by replacing $\Delta E_{\text{bd}+}$ with $\Delta E_{\text{bd}-}$, with the roles of the two states interchanged: on that branch the dark |−2⟩ exciton lies above the bright |−1⟩ one, so that $\Gamma_{b}$ is then the damping of |−2⟩ and the phonon is absorbed rather than emitted. Equation (1) is derived in Section S11, where it appears as Equation (21); the generic notation used there maps onto the present one as |↓⟩→|+2⟩, |↑⟩→|+1⟩, $\Delta_{Z}\to \Delta E_{\text{bd}+}$ and $\Gamma_{↑}\to \Gamma_{b}$. The two key parameters governing the lineshape are: (i) The coupling parameter $a=\left|\mu \right|\sqrt{\Gamma_{e^{\prime }}/\Gamma_{b}}$, where μ is the ratio of the optical matrix elements for the spin‐flip and spin‐conserving channels. It quantifies the relative strength of the resonant spin‐flip pathway with respect to the non‐resonant Brillouin–Mandelstam continuum process. (ii) The phase ϕ, which determines the shape of the resonance profile. Depending on ϕ, one obtains a resonance (cosϕ≈0, sinϕ≈1) or antiresonance (cosϕ≈0, sinϕ≈−1) as well as all possible asymmetric (bipolar) modifications, as depicted in Figure 1e.

Microscopically, ϕ is not a free fitting parameter. Equation (1) can be written exactly as $I/R={\left|1+a\;e^{i\phi }/\left(\varepsilon +i\right)\right|}^{2}$, which identifies $a\;e^{i\phi }/\left(\varepsilon +i\right)$ as the ratio of the resonant to the non‐resonant amplitude. The phase ϕ is the argument of the complex prefactor $\mu D^{\prime }\Gamma_{s}/\left(\Gamma_{b}\Gamma_{e^{\prime }}\right)=\left|\cdots \right|\;e^{i\phi }$, where $D^{\prime }=\hbar \omega_{f}-E_{e^{\prime }}+i\Gamma_{e^{\prime }}$ is the denominator of the non‐resonant pathway and $\Gamma_{s}$ the cross term between the two exciton–phonon couplings; see Section S11. The full relative phase of the two amplitudes contains in addition the energy‐dependent phase of the resonant denominator, arg[1/(ε+i)], which equals $-90^{\circ }$ at resonance. In the outgoing resonance realized here, $|\hbar \omega_{f}-E_{e^{\prime }}|\ll \Gamma_{e^{\prime }}$, one has $D^{\prime }\approx i\Gamma_{e^{\prime }}$; with μ and $\Gamma_{s}$ real, as in Faraday geometry where the exciton eigenstates are pure spin states, this gives $\phi =\pm 90^{\circ }$. For $\phi =-90^{\circ }$ the two amplitudes are then exactly out of phase at ε=0, since $a\;e^{-i\pi /2}/i=-a$; the interference is fully destructive and a symmetric antiresonance results, whereas $\phi =+90^{\circ }$ would give a symmetric resonance. Which sign is realized is set by the sign of $\mu \Gamma_{s}$, which the present model does not evaluate; the observed antiresonance selects $-90^{\circ }$.

In the limit a=0 the resonant spin‐flip channel is absent altogether and Equation (1) reduces to the structureless background $R(\omega_{i},\omega_{f})$. For small but finite a together with $\phi \approx -90^{\circ }$, the profile exhibits a symmetric quasi–Lorentzian antiresonance (AR) in the continuum spectrum whose depth grows linearly as 2a, that is first order in the coupling parameter, whereas the resonant channel on its own contributes only at second order, as $a^{2}$; see Figure 1e. For a≫1, the profile becomes a symmetric Lorentzian function, indicating that the resonant spin‐flip channel dominates over the continuum process. A key distinction from the textbook Fano profile $f\left(\varepsilon ,q\right)={\left(q+\varepsilon \right)}^{2}/\left(1+\varepsilon^{2}\right)$ [31] is that the scattered intensity in Equation (1) reaches zero only under the more restrictive condition asinϕ=−1, which for $\phi =-90^{\circ }$ requires a=1; the intensity then vanishes at ε=−acosϕ. This reflects the more general interference conditions in the spin–phonon scattering geometry.

The width of the resonance is governed by $\Gamma_{b}$, the damping of the upper of the two Zeeman‐split exciton states, which on the (+) branch is the bright exciton, and not by the width of the dark exciton, which does not enter the resonant denominator. We note that the millisecond lifetime quoted in the introduction refers to the radiative and spin lifetime of the ground‐state (*s*‐shell) dark exciton. The *p*‐shell dark exciton addressed here is instead limited by spin‐conserving orbital relaxation into the *s*‐shell on a time scale of tens of picoseconds, and its steady‐state population is maintained by continuous replenishment from the pumped bright state rather than by a long lifetime; see rate‐equation model in Section S12. The same orbital relaxation limits the lifetime of the bright *p*‐shell exciton: for the 10–50 ps assumed in the rate model it corresponds to a homogeneous width $\hbar /\tau_{\text{orb}}=13$–66μeV, whose lower end coincides with the fitted $\Gamma_{b}=13$–18μeV. The identification $\Gamma_{↑}\equiv \Gamma_{b}$, and the expression for a given above, refer to an individual dot. In an ensemble measurement $\Gamma_{b}$ is an effective width. The spectrally narrow laser excites only those dots whose transition is resonant with it, so the relevant distribution is not that of the full ensemble but that of this laser‐selected sub‐ensemble. Within it $\Delta E_{\text{bd}}$ still varies from dot to dot, since the selection acts on the absorption energy and not on the bright–dark splitting itself, and this residual spread contributes to the observed linewidth alongside the homogeneous width set by orbital relaxation. The fitted a is correspondingly an effective ensemble parameter rather than a single‐dot ratio of dampings.

In this Fano‐type interference, the discrete element is the bright–dark exciton transition at the well‐defined energy $\Delta E_{\text{bd}}$, while the continuum is provided by the acoustic phonon density of states, i.e., the quasi‐continuous distribution of phonon modes with different wavevectors k at a given energy. We stress that the phonon spectrum enters our treatment as a continuum: the phonon states are labeled by k and their energies $\hbar \Omega_{k}$ appear inside the energy‐conserving δ‐functions of the scattering rates; see Supporting Information for details. This is what allows the resonance condition $\hbar \Omega_{k}=\Delta E_{\text{bd}}$ to be met at every magnetic field by selecting the appropriate |k|, whereas a discrete spectrum could satisfy it only at isolated fields. The experimental evidence bearing on this is presented in the Discussion.

The energy of the acoustic phonon must match the bright‐to‐dark exciton transition energy, which ranges from tens of μeV to about 400μeV, for varying the magnetic field strength from 0 to 10 T and, correspondingly, the Zeeman splitting. The exciton–phonon coupling is based either on the deformation or piezo‐electricity potential [32, 33]. For semiconductor structures with three‐dimensional confinement having an in‐plane localization size of above 20 nm, like the InGaAs QDs studied, and for energy splittings below 500μeV, the piezo‐electricity is dominant [34, 35]. Since the Fano spin‐flip process is mediated by the l=1 angular component of the exciton–phonon coupling on the transverse branch (as discussed below), we evaluate the relevant spectral density for that branch. The labels l=0 and l=1 used here and in the following denote the angular–momentum components of the exciton–phonon matrix element, not discrete vibrational eigenmodes of the dot.

Writing the phonon plane wave in spherical harmonics, the form factor $\langle \Psi_{f}|e^{ik\cdot r}\left|\Psi_{i}\right\rangle$ contained in $V^{k}$, where $\Psi_{i}$ and $\Psi_{f}$ are the exciton envelopes before and after the scattering, retains only those l permitted by the envelope symmetries of the initial and final exciton states: even l, dominated by l=0, when the two envelopes have equal parity, and odd l, dominated by l=1, when they have opposite parity. The parity is set by the pair of states the phonon connects, not by whether the spin is conserved. The spin‐flip channel is of the odd type, since it is enabled by the spin–orbit admixture of opposite‐parity envelopes discussed in the Discussion; the non‐resonant amplitude that interferes with it connects the intermediate states |e⟩ and $|e^{\prime }\rangle$, which need not have equal parity, and it is its l=1 projection that enters the Fano cross term. The separately observed LA Brillouin–Mandelstam line at 170μeV, discussed below, connects envelopes of equal parity and is dominated by l=0. The phonon energies themselves remain continuous. Following the notation of Ref. [36], we describe the spectral density of the acoustic phonons by $J_{\mathrm{ac}}=AE^{3}\exp\left(-E^{2}/E_{c}^{2}\right)$ with $A=E_{\text{eff}}^{2}/\left(4\pi^{2}\rho \hbar^{4}v_{T}^{5}\right)$, where ρ=5350 kg/$m^{3}$ is the mass density of InGaAs [37] and $v_{T}=3290$ m/s the sound velocity of the transverse acoustic phonons [38, 39]. The cubic low‐energy law is that of a deformation‐potential‐type coupling; for bulk piezoelectric coupling alone the spectral density would be linear in E at low energies [40]. In the spin‐flip channel, however, the odd form factor $\left\langle s|j_{1}\left(kr\right)Y_{1m}|p\right\rangle$ is itself proportional to k at small k, so that its square supplies an additional factor $E^{2}$ and the piezoelectric l=1 channel again acquires the cubic form. We therefore use $J_{\mathrm{ac}}$ as an effective spectral density, with $E_{\text{eff}}$ an effective coupling energy; its absolute value does not enter the shape parameters a and ϕ. The cut‐off $E_{c}=\sqrt{2}\hbar v_{T}/\alpha_{\text{loc}}$ is the form‐factor cut‐off set by the finite carrier localization length $\alpha_{\text{loc}}$ [41, 42]: phonons with $k\;\alpha_{\text{loc}}\gg 1$ oscillate rapidly across the exciton envelope, so their form factor averages to zero and they decouple. With a localization length of the order of the dot radius, $\alpha_{\text{loc}}\approx 20$ nm, this gives $E_{c}\approx 150\;\mu \mathrm{eV}$. The resulting $J_{\mathrm{ac}}$ is accordingly smooth, as expected for a continuous spectrum, and carries no resonant structure. The temperature dependence of the spectral density distribution is considered by the thermal occupation number $N\left(E,T\right)=1/\left\{\exp\left[E/\left(k_{B}T\right)\right]-1\right\}$ [43]. The weighted spectral density $N\left(E,T\right)J_{\mathrm{ac}}$ is shown for a low and high temperature in Figure 1f. Both distributions cover the range of bright–dark exciton splittings accessed in the experiment. Figure 1f shows the absorption weight $NJ_{\text{ac}}$; the emission weight $\left(N+1\right)J_{\text{ac}}$ relevant for phonon emission on the Stokes branch is slightly larger by (N+1)/N, which does not alter the covered range.

### 2.2 Probing Dark Excitons and Negative Exciton Exchange Energy in Polarized QD Emission Spectra

The atom‐like level structure and strong spatial confinement of QDs make them ideal hosts for the spin–phonon Fano resonance described above. We study an ensemble of (In,Ga)As/GaAs QDs which were undoped and rapidly thermally annealed. The rapid annealing allowed for precisely tuning the QD emission energy and reducing the density of defects. The exciton emission of the undoped (In,Ga)As/GaAs QD ensemble measured at 6 K is shown in Figure 2a. Due to shell filling with photocarriers generated in the wetting layer, the different shells contribute to the emission. The tuning of the excitation energy through the ensemble allows for probing excitons from different shells. The *s*‐, *p*‐, *d*‐ and *f*‐shells within the photoluminescence (PL) band are indicated by Gaussian fits (see dashed lines). The shell assignment is supported by the laser‐power dependence of the PL, in which the successive filling of energetically higher shells is resolved, and by PL excitation spectroscopy, which locates the wetting‐layer edge at about 1.490 eV and thereby fixes the energy scale of the confined states (Figure S5). We note that the weaker *d*‐ and *f*‐shell components remain the least well constrained. The shell dependence reported below follows from the systematic variation, and sign reversal of $\delta_{0}$ with excitation energy, which is independent of how the PL band is partitioned. Resonantly exciting the *p*‐shell at $E_{\text{exc}}=1.4571$ eV (laser linewidth <4μeV) and monitoring the QD emission within the spectral range from −500 to +500μeV centered at $E_{\text{exc}}$ yield the spectra shown in Figure 2b. Hereby, an external magnetic field is applied along the QD growth z‐axis. In this longitudinal magnetic field (Faraday) geometry, clear Fano ARs with coupling parameters of $a_{\text{bd+}}<0.12$ and phases $\phi_{\text{bd+}}\approx -90^{\circ }$ are present. Their spectral positions $E_{\text{Fano}}$ are specified with respect to the excitation energy $E_{\text{exc}}$. The Fano ARs thus lie below (Stokes) as well as above (anti‐Stokes) the excitation energy. Only when the circular polarization of the incident laser light is equal to that of the emission, the Fano AR appears in the spectra; see also Figure 3e. For the z($\sigma^{+}$, $\sigma^{+}$)$z^{\prime }$ polarization configuration, the Fano AR is at the Stokes side. It points out that the bright |+1⟩ *p*‐shell exciton is involved and on account of the Fano‐like quantum interference, the Fano AR represents the dark |+2⟩ exciton state, as described within the theoretical framework. The addressing of the |−1⟩ exciton with $\sigma^{-}$ polarized light induces only at the anti‐Stokes side a Fano AR displaying the |−2⟩ dark exciton state. This underlines not only the participation of the dark exciton state |+2⟩ (|−2⟩) in the optical process with the $\sigma^{+}$ ($\sigma^{-}$) copolarized configuration, but also the presence of the |−1⟩ *p*‐shell exciton as lowest energy state. The access to the dark exciton as well as the exciton level hierarchy are thus manifested in distinct polarization selection rules for state‐selective resonant excitation.

**FIGURE 2:**
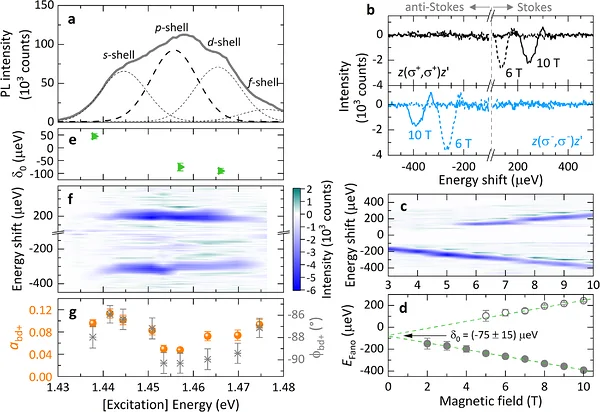
Polarized QD emission spectra for probing dark excitons with negative exciton exchange energies measured in Faraday geometry (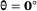) at T = 6 K. (a) PL spectrum of undoped InGaAs/GaAs QD ensemble excited at 1.533 eV. The contributions of the different QD shells are marked by Gaussian fits (dashed lines). (b) Fano antiresonances in the circularly polarized QD emission excited at 1.4571 eV, addressing the *p*‐shell excitons. The energy scale is referred to the laser excitation energy, which corresponds to the zero energy shift. Magnetic‐field evolution of the (c) Fano AR spectra and (d) spectral positions of the Fano ARs; panels c and f share the intensity scale. The extrapolations of $E_{\text{Fano}}$ to 0 T (dashed lines) indicate a negative exciton exchange energy $\delta_{0}$. The open (closed) circles denote $E_{\text{Fano}}$ at the Stokes (anti‐Stokes) side. (e) $\delta_{0}$ is obtained for exciting excitons at different QD shells; these data were measured in a tilted geometry with 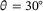 to allow for evaluating $\delta_{0}$ at the *s*‐shell. Excitation energy dependence of the (f) Fano AR spectra across the QD shells and (g) fit parameters $a_{\text{bd+}}$ and $\phi_{\text{bd+}}$ for the Fano process involving the |+1⟩ and |+2⟩ excitons; B = 8 T. In panel f the spectra at positive (negative) energy shifts were measured in $z\left(\sigma^{+},\sigma^{+}\right)z^{\prime }$ [$z\left(\sigma^{-},\sigma^{-}\right)z^{\prime }$] configuration.

**FIGURE 3:**
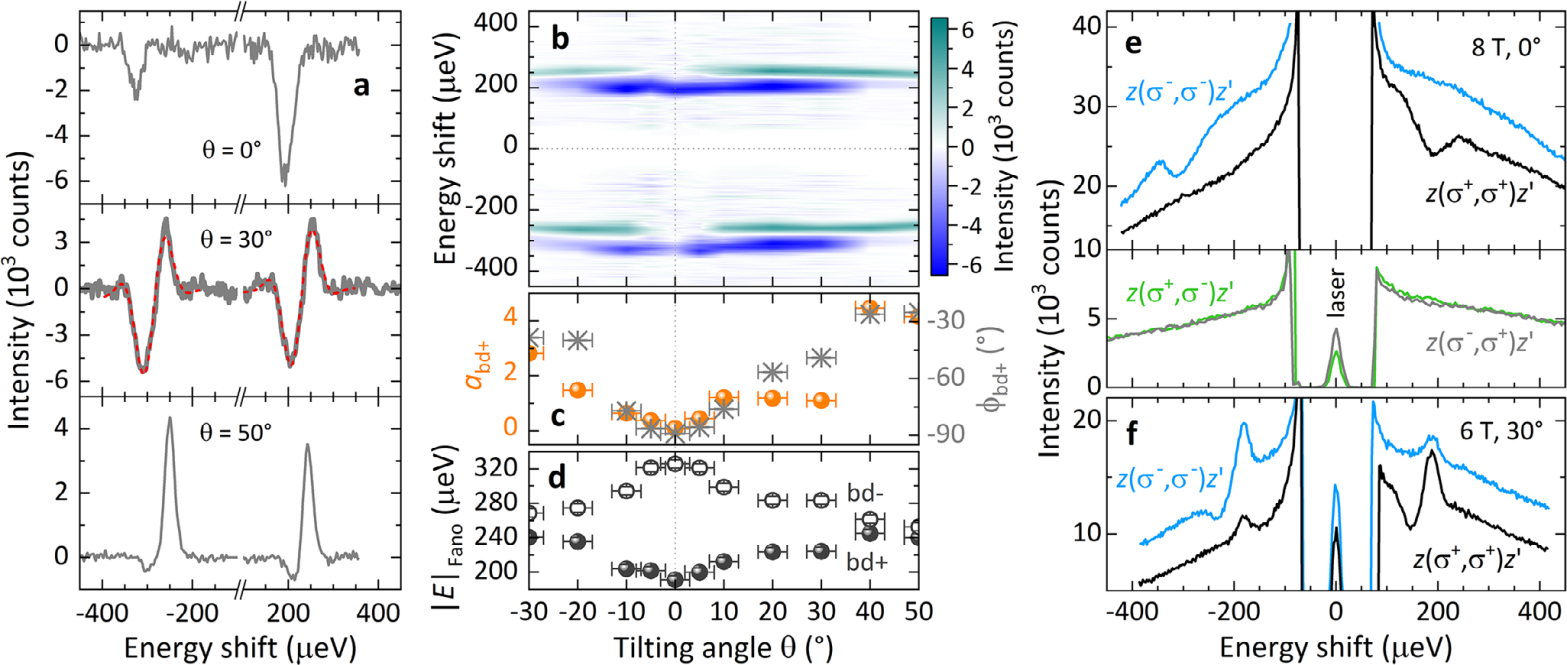
Tuning the Fano resonance through the magnetic field orientation. (a) Fano resonance spectra detected in three different magnetic‐field geometries; B = 8 T. The spectra at positive (negative) energy shifts were measured in $z\left(\sigma^{+},\sigma^{+}\right)z^{\prime }$ [$z\left(\sigma^{-},\sigma^{-}\right)z^{\prime }$] configuration. The red dashed lines in the middle panel mark the fitting curves after Equation (1) with specific a and ϕ. Dependence of the (b) Fano resonance spectra, (c) parameters $a_{\text{bd+}}$ and $\phi_{\text{bd+}}$ and (d) spectral positions on the tilting angle θ. The parameters of the Fano ARs clearly differ from those of the bipolar shaped Fano resonances obtained for 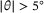. Polarization‐resolved QD emission demonstrates (e) Fano ARs in Faraday geometry and (f) bipolar shaped Fano resonances and Gaussian‐like peaks (see text) at 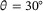.

The shells overlap spectrally in the ensemble PL, but the scattering is not averaged over the whole distribution: with a laser linewidth below 4μeV only a narrow interval of transition energies is addressed. This does not by itself isolate a single orbital shell, since dots of different size can bring different shell transitions into resonance at the same energy; the evidence that the selection is nevertheless shell‐sensitive is the measurement itself, as $\delta_{0}$ varies systematically and changes sign across the band (Figure 2e) rather than taking a single ensemble‐averaged value, so that the shell composition of the excited sub‐ensemble must change as the excitation energy is tuned. A contribution from defect or trap states can also be excluded, since such scattering would obey neither the circular polarization selection rules nor the field dispersion of the exciton Zeeman splitting.

We now turn to a quantitative consequence of these polarization‐resolved spectra: the magnetic‐field dependence of the Fano AR positions provides direct access to the exciton exchange energy $\delta_{0}$, and reveals that its sign and thus the bright–dark level hierarchy depends on the QD shell. This shell‐dependent sign reversal is one of the central results of the present work and has so far escaped detection by conventional PL spectroscopy.

Changing the external magnetic field strength shifts the spectral position of the Fano ARs and, correspondingly, the dark exciton states, as exemplarily shown for B = 6 T and 10 T in Figure 2b. Their detailed magnetic‐field dependences are depicted in Figure 2c, and the spectral positions as function of B are presented in Figure 2d. $E_{\text{Fano}}\left(B\right)$ at the Stokes (anti‐Stokes) side indicates the energy difference between the bright and dark excitons $\Delta E_{\text{bd}+}=E_{X,+1}-E_{X,+2}$ ($\Delta E_{\text{bd}-}=E_{X,-1}-E_{X,-2}$), following the same field dependence as in Figure 1b. The Stokes and anti‐Stokes Fano ARs deviate significantly in energy from each other; for instance, at 10 T the Fano AR at the Stokes (anti‐Stokes) side lies at about 250μeV (−390μeV). A linear fit to the measured field dependence of both branches returns a slope of (31.5±0.7)μeV/T, in agreement with the electron Zeeman splitting $|g_{z}^{\mathrm{el}}|\mu_{B}=31.8\;\mu \mathrm{eV}$/T expected for a pure electron spin flip between |+1⟩ and |+2⟩ (and between |−1⟩ and |−2⟩), and a zero‐field offset of −71μeV that agrees with the value of $\delta_{0}$ obtained below from the extrapolation of both branches. The measured dispersion thus confirms directly that the Fano resonance tracks a transition in which only the electron spin is reversed, with an electron g‐factor equal to its s‐shell value within the uncertainty.

Following the magnetic field evolutions down to 0 T (dashed lines in Figure 2d), an offset of (−75±15)μeV becomes apparent. This zero‐field offset equals the axially symmetric part $\delta_{0}$ of the exchange interaction between the bright and dark excitons. The negative $\delta_{0}$ leads to a *p*‐shell exciton fine structure in which the optically bright |−1⟩ exciton is lowest in energy. Within the envelope function model of Ref. [44], the *p*‐shell exciton states are composed of *p_x_* and *p_y_* envelope functions lying in the lateral QD *xy*‐plane, and the interplay of Coulomb interaction and valence band mixing leads to strongly mixed spin configurations whose exchange splitting $\delta_{0}$ can differ in sign from the *s*‐shell value. These mixed *p*‐shell states are most probably involved in the Fano process. While the sign and value of $\delta_{0}$ are only negligibly varied by the magnetic field alignment with respect to the QD growth axis, details are given in the Figure S1, they significantly depend on the excitation energy. As presented in Figure 2e, we obtain $\delta_{0}=\left(-90\pm 10\right)\;\mu \mathrm{eV}$ at the *d*‐shell and for $E_{\text{exc}}=1.438$ eV at the low‐energy flank of the *s*‐shell it becomes positive and takes the value $\delta_{0}=\left(45\pm 10\right)\;\mu \mathrm{eV}$. Going from the *s*‐shell to energetically higher shells, the variation in $\delta_{0}$ including a sign reversal demonstrates that the level hierarchy of the bright and dark excitons is inverted; only at the *s*‐shell does the energetically lowest exciton remain dark.

Focusing on the excitation energy dependence of the Fano AR, Figure 2f shows us that the Fano AR is significantly pronounced for resonantly probing the *p*‐shell states of the QDs. The depth of the Fano AR (blue colored intensity scale) decreases continuously for both increasing the excitation energy from the *p*‐shell to the *f*‐shell exciton resonances of the QDs as well as decreasing $E_{\text{exc}}$ below the center of the *s*‐shell. At its low‐energy flank the spectra contain only a smooth background. Moreover, the spectral position of the Fano AR slightly shifts toward low energies for $E_{\text{exc}}$ rising from 1.438 to 1.480 eV. Considering this excitation energy range, $\delta_{0}$ and the exciton Zeeman splitting decreases; it is expected that the electron spin splitting is reduced by about 10% (30 μeV at 8 T) [29, 45]. It is worthwhile to mention that in a tilted magnetic field geometry the Fano resonance is also particularly pronounced at the *p*‐shell; see Figure S2.

Now, for providing a more quantitative analysis of the Fano resonance shape, we model it by our Fano profile of Equation (1), yielding the parameters a and ϕ. As presented in Figure 2g, the Fano AR at the Stokes side in longitudinal geometry (tilting angle 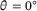) is characterized by $a_{\text{bd+}}$ values of 0.04±0.01 at the center of the *p*‐shell emission, with $\phi_{\text{bd+}}={\left(-90.1\pm 0.5\right)}^{\circ }$ confirming a nearly pure antiresonance profile. For excitation energies deviating from the *p*‐shell center, $a_{\text{bd+}}$ increases slightly to about 0.1, while $\phi_{\text{bd+}}$ remains close to −π/2 across all QD shells. The values of $a_{\text{bd+}}$ in Figure 2g require care in interpretation. Since a is a ratio of the two scattering amplitudes, a small value signifies that the non‐resonant continuum dominates, not that the bright–dark coupling is weak. Accordingly, the depth of the antiresonance, which scales as $(2a-a^{2})R$, is largest at the center of the *p*‐shell because the background R peaks there, even though $a_{\text{bd+}}$ is smallest there. The rise of $a_{\text{bd+}}$ on either flank cannot be attributed to the number of dots excited, which multiplies both amplitudes alike and cancels from their ratio; it resides in the $a=\left|\mu \right|\sqrt{\Gamma_{e^{\prime }}/\Gamma_{b}}$ prefactor, whose individual factors cannot be separated by these data.

### 2.3 Tunability of the Fano Resonance with Magnetic Field Orientation, Laser Power, and Temperature

Leaving the longitudinal field geometry and tilting the magnetic field direction by the angle θ with respect to the QD growth axis yields bipolar‐shaped Fano resonances, as shown in Figure 3a. The intensities of the Fano resonances involving the |+1⟩→|+2⟩ and |−1⟩→|−2⟩ transitions are mapped angle‐resolved in Figure 3b with θ varied from $-30^{\circ }$ to $+50^{\circ }$. While at 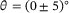 the Fano AR is present, a pronounced bipolar Fano shape is visible from about $-30^{\circ }$ up to about $35^{\circ }$. For even larger tilting angles a Lorentzian‐like shaped peak is observed. At increasing angles θ, the coupling parameter $a_{\text{bd+}}$ grows from values below 0.1 at $\theta =0^{\circ }$ to values exceeding 4 at $\theta >40^{\circ }$, while the phase $\phi_{\text{bd+}}$ evolves from $-90^{\circ }$ toward approximately $-30^{\circ }$, as presented in Figure 3c. Furthermore, the spectral position $E_{\text{Fano}}$ shifts with increasing θ to higher (lower) energies for the Fano resonance at the |+2⟩ (|−2⟩) dark exciton state, as shown in Figure 3d. In strongly oblique geometries, the Fano resonance energies converge to each other. This convergence agrees well with the angular dependence of the bright and dark exciton energy differences: For 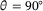 and 8 T, a calculation leads to $\Delta E_{\text{bd}+}\approx \Delta E_{\text{bd}-}\approx 245$ μeV; see Supporting Information for details.

The Fano AR is changed to the bipolar resonance shape by the mixing of the electron spin basis eigenstates |+1/2⟩ and |−1/2⟩ of both the bright as well as the dark exciton. In an oblique magnetic field geometry with B=B(sinθ,0,cosθ), the off‐diagonal Zeeman term $\frac{1}{2}\mu_{B}g_{⊥}^{\text{el}}B\sin\theta$ couples the |+1/2⟩ and |−1/2⟩ spin states [46, 47]. The resulting mixing, whose strength is governed by the ratio of this off‐diagonal energy to the relevant level splitting, increases the resonant spin‐flip amplitude relative to the non‐resonant continuum, and it is this change in the balance of the two scattering channels that the rise of $a_{\text{bd+}}$ with θ reported above reflects. The anti‐Stokes parameters $a_{\text{bd}-}$ and $\phi_{\text{bd}-}$ show similar values, confirming that the same phonon coupling governs both processes.

In an inhomogeneous ensemble the excitation always finds a subset of dots in outgoing resonance, so $D^{\prime }\approx i\Gamma_{e^{\prime }}$ is maintained at every excitation energy; Figure 2g bears this out, $\phi_{\text{bd+}}$ remaining at $-90^{\circ }$ across the whole PL band. Tilting the field mixes the electron spin states of both the bright and the dark exciton. This modifies the complex ratio of the two interfering amplitudes: the optical and phonon matrix elements entering μ and $\Gamma_{s}$ become admixtures of those of the unmixed states, and further intermediate states with different complex denominators are admitted. The net effect is a shift of ϕ away from $-90^{\circ }$ that turns the antiresonance into the bipolar profile of Figure 3a; a quantitative evaluation of this change is beyond the present model. Because the level splitting that governs the mixing is shell dependent through $\delta_{0}$, ϕ additionally varies with excitation energy in the tilted geometry (Figure S2c) while remaining constant in the untilted one.

Besides the tailoring of the Fano profile by specifically orienting an external magnetic field, Figure 3f presents a further intriguing detail observed in tilted geometries: Gaussian‐like peaks are detected in cocircular polarizations at the energy side opposite to that exhibiting the Fano resonance, i.e., in $z\left(\sigma^{+},\sigma^{+}\right)z^{\prime }$ [$z\left(\sigma^{-},\sigma^{-}\right)z^{\prime }$] at the anti‐Stokes [Stokes] side. At these energy shifts, the resonance condition for the bright–dark exciton spin‐flip transition is not fulfilled, so the resonant scattering pathway does not contribute. Only the non‐resonant Brillouin–Mandelstam process remains, and the quantum interference underlying the Fano effect does not occur. The scattering gives rise to symmetric emission lines. Their Gaussian‐like shape reflects the inhomogeneous broadening of the QD ensemble, since in the absence of the Fano interference no intrinsic lineshape asymmetry or resonant narrowing occurs. The Stokes and anti‐Stokes energy shifts of these peaks are equal and correspond to the electron Zeeman splitting $\mu_{B}\left|g_{z}^{\text{el}}B\right|$ with negligible excitonic exchange energies; details on the magnetic field dependence are given in the Figure S4. Due to the fast hole spin‐relaxation time in tilted magnetic field, the electron and hole spins are only weakly correlated. Accordingly, the exchange interactions do not play a role [48].

These Gaussian‐like peaks are conventional spin‐flip Raman scattering, and the contrast with them shows what the Fano channel adds. They appear at the bare electron Zeeman splitting and only in tilted geometry, where the resonant pathway is switched off; the Fano feature instead appears at $\Delta E_{\text{bd}}$ and so includes the exchange energy $\delta_{0}$. A Raman line requires the spin‐flip scattering to exceed the background on its own, whereas the antiresonance appears through the interference term, which is first order in a rather than second order as the resonant channel alone would be. This is why the bright–dark transition is accessible in Faraday geometry, where no spin‐flip Raman line is resolved, and why the method does not require the symmetry breaking that conventional spin‐flip Raman scattering needs.

The bipolar shape of the Fano resonance becomes further tuned by the laser power $P_{\text{exc}}$, as demonstrated in Figure 4a. The coupling parameter $a_{\text{bd+}}$ steadily decreases from about 5 to 1 (factor 5) when $P_{\text{exc}}$ is raised from 0.1 mW to about 50 mW; see spheres in Figure 4b. This enhanced bipolar Fano profile is accompanied by a linearly intensified (factor 600) background intensity $I_{\text{back}}$ of the emission spectra; see squares in Figure 4b. On the one hand, the laser light drives the bright–dark exciton transition indirectly by generating bright excitons in the *p*‐shell. On the other hand, the optical excitation leads to spectrally broad emission scattered inelastically by acoustic phonons, which resembles the acoustic phonon sideband of the bright excitonic zero‐phonon transition [49]. The intensity enhancement of this background suggests that the bright–dark exciton transition becomes more strongly coupled to the acoustic phonon continuum. Both trends can be understood as consequences of the excitation dependence of the populations and dampings rather than of the phonon occupation; since the treatment underlying Equation (1) is linear in the excitation, we propose the following scenario . The background $I_{\text{back}}$, which we identify with R, contains a contribution proportional to the steady‐state dark population; this grows with the pump rate while the shells are unsaturated and accounts for the rise of $I_{\text{back}}$ by three orders of magnitude. The coupling parameter is the amplitude ratio $a=\left|\mu \right|\sqrt{\Gamma_{e^{\prime }}/\Gamma_{b}}$, from which the population cancels, so its decrease requires $\Gamma_{b}$ to grow faster with carrier density than $\Gamma_{e^{\prime }}$, as expected once carrier–carrier scattering adds to the spin relaxation. Above about 50 mW the wetting‐layer emission dominates and the shell occupancies, and with them $N_{d}$ and the carrier‐density‐driven growth of $\Gamma_{b}$, saturate (Figure S5a). $I_{\text{back}}$ levels off accordingly, whereas $\Gamma_{e^{\prime }}$ continues to grow with the wetting‐layer carrier density, so that the ratio $\Gamma_{e^{\prime }}/\Gamma_{b}$ reverses its trend and $a_{\text{bd+}}$ turns and rises again.

**FIGURE 4:**
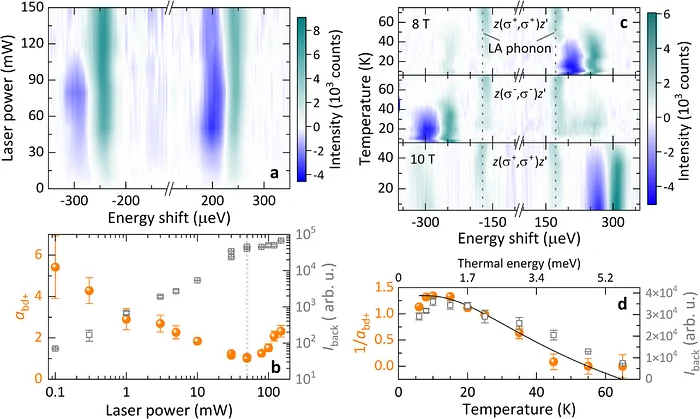
Modifying the bipolar shaped Fano resonance of the *p*‐shell exciton by the laser power and temperature at 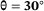. (a) Laser power dependence of the Fano resonance spectra whose parts at positive (negative) energy shifts were detected in $z\left(\sigma^{+},\sigma^{+}\right)z^{\prime }$ [$z\left(\sigma^{-},\sigma^{-}\right)z^{\prime }$] configuration at 8 T and 6 K. (b) Tuning of the factor $a_{\text{bd+}}$ and spectral background intensity $I_{\text{back}}$ by the laser power $P_{\text{exc}}$. The scales of $P_{\text{exc}}$ and $I_{\text{back}}$ are logarithmic. To demonstrate the full range of $a_{\text{bd+}}$ values depending on $P_{\text{exc}}$, the oblique magnetic field geometry with $\theta =30^{\circ }$ was chosen. (c) The temperature evolution reveals intensity and shape variations of the Fano resonance as well as the appearance of the LA phonon line. (d) Dependence of the inverse coupling factor and background intensity on the temperature. The behavior of $1/a_{\text{bd+}}$ is described empirically by $\frac{1}{a_{\text{bd+}}}=A_{0}-A_{1}\exp\left(-\frac{E_{T}}{k_{B}T}\right)$, with the dimensionless parameters $A_{0}=1.3$ and $A_{1}=4.1$ and an empirical thermal scale $E_{T}=\left(5.2\pm 0.3\right)$ meV.

In Figure 4c, the impact of direct changes in the lattice temperature on the Fano resonance is presented. The bipolar Fano resonance is observed at temperatures ranging from 6 K to about 40 K, both for the |+1⟩→|+2⟩ and |−1⟩→|−2⟩ transitions. $a_{\text{bd+}}$ lies slightly below 1 ($1/a_{\text{bd+}}>1$) until 20 K and grows strongly above 20 K so that the inverse coupling parameter $1/a_{\text{bd+}}$ tends to zero, as depicted in Figure 4d. Moreover, the background intensity (gray squares) exhibits a temperature dependence similar to that of $1/a_{\text{bd+}}$, i.e., $I_{\text{back}}$ increases or, respectively, remains at a high level up to 20 K and shrinks for rising temperatures until 65 K. The similar temperature behavior of $1/a_{\text{bd+}}$ and $I_{\text{back}}$ highlights the relevance of the acoustic phonons participating in the Fano process. For elevated temperatures, the disappearance of the Fano resonance may additionally be related to an enhanced quadratic electron–phonon coupling, which was identified as an important thermal dephasing mechanism for *p*‐shell excitons [50]. The exciton PL remains stable at increasing temperatures; see Figure S7. Thermally induced red‐shifts in the PL energies are small (from 5 to 50 K they amount to about 2.8 meV) so that their influence on the excitation and detection conditions of the Fano resonance, including particularly *p*‐shell excitons, is negligible.

The thermal evolution of $1/a_{\text{bd+}}$ is described empirically by an exponential (Arrhenius‐like) modeling, see black curve in Figure 4d, which yields a characteristic energy of (5.2±0.3) meV, equivalent to about 60 K. With $A_{0}=1.3$ and $A_{1}=4.1$ the fitted expression crosses zero near 52 K and becomes negative above it, which is unphysical for an inverse amplitude ratio, so the form should be regarded as an interpolation over the measured range and not extrapolated beyond it. The loss of the bipolar profile is neither a depopulation of the dark state nor a shortage of resonant phonons. As already underlined, the dark population enters both amplitudes as a common prefactor and cancels from a and ϕ, and the phonon occupation at the bright–dark splitting rises with temperature rather than falling: at the measured $\Delta E_{\text{bd}+}$ of about 190μeV it grows from N=2.2 at 6 K to N=26 at 60 K, and the weighted spectral density of Figure 1f rises correspondingly, its maximum moving only from 144 to 149μeV while its peak value increases by one order of magnitude, which is why the two curves are plotted on scales differing by a factor of ten. The evolution must therefore lie in the amplitudes themselves. A thermally increasing broadening of the intermediate states acts in the observed direction, since $a\propto \Gamma_{e^{\prime }}^{1/2}$ while the background scales inversely with $\Gamma_{e^{\prime }}$, and both $1/a_{\text{bd+}}$ and $I_{\text{back}}$ do fall above 20 K. Quantitatively, however, this scaling would give a far steeper decrease of $I_{\text{back}}$ than the factor of about six observed, so we quote the thermal behavior as an empirical result. Nevertheless, it is worthwhile to underline that a thermal energy of $k_{B}T\approx 5$ meV exceeds the bright–dark exciton splitting measured at 8 T by a factor of about 16 for $|\Delta E_{\text{bd}-}|$ and about 27 for $\Delta E_{\text{bd}+}$. The interference therefore remains spectroscopically resolvable up to about 40 K, at thermal energies well above the bright–dark exciton splitting itself. We note that this comparison of energy scales is not in itself a criterion for coherence, since $k_{B}T$ and $\Delta E_{\text{bd}}$ do not enter the lineshape on the same footing; what the data establish is that the interference survives to that temperature.

A further interesting observation is the appearance of a peak at about 170 μeV in cocircular polarizations at T>20 K; see Figure 4c, in which the temperature dependence of this feature is shown at fixed magnetic field: it is absent at the base temperature, emerges above about 20 K, and gains intensity as the acoustic phonon occupation grows, in contrast to the Fano resonance, whose bipolar contrast is lost over the same range. Quantitatively, the integrated intensity of the line follows the acoustic phonon occupation over the whole range in which it is resolved: on the anti‐Stokes side it scales as $N(E_{\text{LA}},T)$ and on the Stokes side as $N(E_{\text{LA}},T)+1$, and the ratio of the two equals $\exp(E_{\text{LA}}/k_{B}T)$ within the experimental uncertainty (Figure S17). Hence, this feature is related to inelastic scattering by a thermally populated acoustic mode. Its spectral position does not shift with the external magnetic field, it is even present at zero field (see Figure S8), and its Stokes and anti‐Stokes intensities are similar. Moreover, the peak is observed for resonantly probing the *s*‐ and *p*‐shells of the InGaAs QD ensemble. We attribute this peak to Brillouin–Mandelstam scattering by a longitudinal acoustic (LA) phonon whose coupling to the s‐ and p‐shell excitons proceeds through the radial, l=0 component of the form factor [51, 52]. The preservation of the light helicity confirms that the l=0 component dominates, whose spherical symmetry does not disturb the polarization properties [53]. The phonon involved is a propagating mode whose wavevector is fixed by momentum conservation in the backscattering geometry rather than by the dot size, so its energy is given by the Brillouin relation $E_{\text{LA}}\approx 2E_{\text{exc}}\left(\frac{n^{\prime }}{c}\right)v_{L}\sin\frac{\varphi }{2}$ [54], where $n^{\prime }=3.6$ is the refractive index of InGaAs [55] and $v_{L}=4670$ m/s the longitudinal sound velocity; see Supporting Information for details. In our experiment, the optical axes of the incident and detected light are rotated approximately $10^{\circ }$ from each other, leading to a scattering angle $\varphi ∡(k_{\text{exc}},k_{\text{det}})$ of 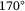. For $E_{\text{exc}}=1.455$ eV, $E_{\text{LA}}$ amounts to (163±9) μeV, in agreement with the measured shift of about 170μeV. This line independently demonstrates that bulk‐like acoustic Brillouin–Mandelstam scattering occurs in this QD ensemble: it is measured in a cocircular configuration, is present at zero magnetic field and does not shift with B, so the spin‐flip channel plays no role in it. It does not, however, identify the phonon mediating the Fano process, which is drawn from the continuous transverse branch at the wavevector for which $\hbar \Omega_{k}=\Delta E_{\text{bd}}$, and which is inferred from the field dispersion and the coupling symmetry rather than resolved spectroscopically. It is remarkable that both the LA phonon line, observed only at higher temperatures, and the Fano resonance are simultaneously detected and in specific cases they even overlap with each other.

## 3 Discussion

*Which acoustic phonons mediate the Fano interference, and are they confined?* The acoustic phonons of the InGaAs/GaAs system are bulk‐like: the acoustic impedance ρv of the two materials differs by less than 2% (mass densities 5350 and 5310 kg/$m^{3}$, transverse sound velocities 3290 and 3368 m/s), so that strongly confined discrete modes are not expected, i.e., a vibration localized on the dot would leak rapidly into the surrounding material [56, 57]. The continuum labeled by k is therefore what plays the role of the Fano continuum against which the discrete bright–dark transition interferes. Two observations support this. First, the Fano energy follows $\Delta E_{\text{bd}}\left(B\right)$ continuously from about 100 to about 400μeV (Figure 2c,d), whereas a discrete spectrum would produce the resonance only at isolated fields. Second, the spin‐conserving LA line at 170μeV, the one acoustic feature accessible independently of the spin‐flip channel, has a width of about 20μeV, well above the laser linewidth and the spectrometer resolution. Inhomogeneous broadening can only widen it, so this is an upper bound on the homogeneous width and the coherence length $\hbar v_{L}/\Gamma_{\text{LA}}≳140$ nm a lower bound; it exceeds the dot diameter of (40±4) nm threefold. A confined vibration would be leaky and hence broad, so a line this narrow is hard to reconcile with confinement. Since both acoustic branches experience the same elastic mismatch, this supports the bulk‐like character of the acoustic spectrum as a whole, though the transverse branch relevant to the Fano process is not itself resolved.

The interference channel follows from the same picture. Two amplitudes interfere only if they leave the system in the same final state and project onto the same angular channel: summing over the phonon directions, components of different l drop out by orthogonality. The interference therefore occurs within a single channel of the transverse continuum, the resonant amplitude being dominated by l=1 (see below) and the interfering non‐resonant amplitude carrying an l=1 projection, which it may because the intermediate states connected by the phonon need not have equal parity. The longitudinal 170μeV line belongs to a different branch; it demonstrates that bulk‐like Brillouin–Mandelstam scattering occurs in this ensemble, but it does not identify the phonons of the Fano channel. What distinguishes the two channels is thus not confinement but the angular symmetry of the exciton–phonon coupling.

*Which states does the interference connect, and what makes the spin flip allowed?* Interference requires both pathways to share initial and final states, so the cycle is closed and must start from a populated state. At 8 T the |+1⟩ bright exciton lies above |+2⟩: a cycle starting from the dark state returns to it by emitting a phonon and appears on the Stokes side under $\sigma^{+}$ excitation, whereas one starting from the bright state would require absorption and appear on the anti‐Stokes side. For the |−1⟩/|−2⟩ pair the ordering, and hence the side, is reversed. Both predictions are precisely observed (Figure 2b,c).

Which shell the dark exciton belongs to is fixed the same way. *p*‐shell excitons relax rapidly to the *s*‐shell, and the *s*‐shell dark reservoir is far more populated (Supporting Information), but it does not contribute: both amplitudes carry the resonant denominator $\hbar \omega_{i}-E_{e}+i\Gamma_{e}$, so only states resonant with the laser light energy participate. Decisively, the exchange energy extrapolated to zero field changes sign across the ensemble, from (+45±10)μeV at the *s*‐shell flank to (−75±15) and (−90±10)μeV at the *p*‐ and *d*‐shells (Figure 2e); a common *s*‐shell origin would give a single value throughout.

Decomposing the form factor $\langle \Psi_{f}|e^{ik\cdot r}\left|\Psi_{i}\right\rangle$ into spherical harmonics, the l=0 component connects envelopes of equal parity and conserves the exciton spin. It is not the background against which the Fano resonance forms: that background is the non‐resonant amplitude within the same transverse l=1 channel as the resonant one. The line at 170μeV is a separate, momentum‐conserving Brillouin–Mandelstam process and does not enter the interference.

The bright and dark *p*‐shell excitons differ in the electron spin and share the same envelope parity, so the spin flip is not mediated by the deformation‐potential or piezoelectric coupling alone: both are spin diagonal, and for equal parity the odd‐l components vanish. It proceeds instead through the spin–orbit admixture of the confined states. Writing $|\tilde{p},↑\rangle =\left|p,↑\right\rangle +\alpha \left|s,↓\right\rangle +\cdots$, the spin‐diagonal interaction couples the admixed component α|s,↓⟩ of $|\tilde{p},↑\rangle$ to |p,↓⟩, the principal component of the spin‐flipped partner state $|\tilde{p},↓\rangle$, so the effective bright–dark matrix element is proportional to α and to $\left\langle s|j_{1}\left(kr\right)Y_{1m}|p\right\rangle$. The spin–orbit admixture flips the spin; the odd‐l envelope factor makes the resulting element non‐vanishing. We therefore write $V_{\text{bd}}^{k}$ as an effective spin–phonon matrix element generated by this mixing, and it is its l=1 component that carries the required unit of angular momentum [58, 59]. Since the l=1 radial factor $j_{1}\left(kr\right)$ vanishes at the dot centre and peaks near its perimeter, it overlaps efficiently with the pair of opposite‐parity envelopes that the spin–orbit admixture connects; the corresponding distributions are compared in Figures S9 and S10. The form factor does not, however, carry the dependence on the addressed shell: by hermiticity the same s–p cross integral appears whether the *s*‐ or the *p*‐shell exciton is excited. Any intrinsic shell dependence must reside in the admixture amplitude α, which is governed by the spin–orbit matrix elements between neighboring shells and by their energy separations, and whose evaluation lies beyond the scope of the present analysis. The prominence of the antiresonance at the *p*‐shell in Figure 2f does not directly reflect the coupling strength: the depth scales as $(2a-a^{2})R$, and the non‐resonant background R follows the inhomogeneous distribution of resonantly excited dots, which peaks at the *p*‐shell. Consistently, $a_{\text{bd+}}$ itself is smallest there (Figure 2g). The same reasoning applies to the electron–confined‐phonon coupling analyzed for wires and slabs [60, 61], where a genuine elastic mismatch does produce structure in the spectral density—a structure that is absent here, consistent with the smooth $J_{\mathrm{ac}}$ of Figure 1f, and none of which invokes any quantization of the phonon spectrum.

Since the initial state already contains an electron–hole pair, the absorbed photon creates a two‐exciton configuration, so that |e⟩ and $|e^{\prime }\rangle$ may be identified with biexcitonic states having one exciton in the *p*‐shell and the second in the *s*‐ or *p*‐shell, differing by the spin of the added carrier. Within this interpretation the spin‐singlet ground‐state biexciton is not accessible: it carries zero angular‐momentum projection and cannot be reached from |+2⟩ by a single $\sigma^{+}$ photon. The two‐photon step |+2⟩→|+1⟩ changes the projection by one unit and would be forbidden in an axially symmetric dot; it is allowed here because the *p*‐shell states are built from $p_{x}$ and $p_{y}$ envelopes whose Coulomb and valence‐band mixing renders the projection not well defined. That same $p_{x}$/$p_{y}$ composition makes the l=1 component of the coupling non‐vanishing on the return step, so the selection rule discussed here and the angular decomposition analyzed above are two aspects of one mechanism.

*Why do the Stokes and anti‐Stokes Fano resonances appear inverted, and what does this tell us about the underlying interference?* The observation of the dark exciton and, in turn, the spin–phonon Fano resonance mechanism are sensitive to the energy and spin differences between the bright and dark exciton states and to the shapes of the electron and phonon wavefunction distributions. The energetic dependence also manifests itself in a broadening of the Fano AR (Figure 2b), for increasing magnetic field strength, on account of a dispersing Zeeman splitting of the exciton states as well as a shortening of the exciton spin lifetime. Fitting Equation (1) to the spectra of Figure 2c returns a damping $\Gamma_{b}$ that grows from about 13μeV at 6 T to about 18μeV at 10 T on the Stokes branch, over the field range in which its width can be extracted reliably, and lies between 14 and 19μeV on the anti‐Stokes branch across the whole range. The bipolar Fano resonances involving the |±2⟩ dark exciton states appear inverted to each other. This inversion does not arise from different coupling parameters: the fitting yields similar values of a and ϕ for the Stokes and anti‐Stokes processes. Instead, the apparent shape inversion results from the reduced energy ε changing sign on opposite sides of the resonant bright–dark exciton splitting $\Delta E_{\text{bd}}$. Consequently, the term εcosϕ in Equation (1) changes sign and reverses the asymmetry of the bipolar profiles. The reflection and transmission geometries yield identical bipolar shapes (see Figure S11), consistent with the acoustic phonons being mainly formed within the QD plane perpendicular to the light propagation and the magnetic field direction (in Faraday geometry) [62, 63].

*Could the Fano resonance be an artifact of charged‐exciton resonances or absorption features rather than a spin–phonon interference?* For resonantly exciting InGaAs QDs charged with a single electron, neither a Fano AR in Faraday geometry nor a bipolar shaped Fano resonance in tilted geometries are observed; see Figure S12. Only a Gaussian shaped line is detected, and its spectral position follows the electron Zeeman splitting. In the singly charged dots whose PL energy is comparable to that of the neutral QDs studied, negatively charged excitons (negative trions) are probed. Hereby, isotropic and anisotropic exchange interactions between the carriers of the trion dominate over couplings with acoustic phonons [29, 64, 65]. Thus, this control experiment on the charged QDs underlines that in the neutral dots the dark excitons and acoustic phonons govern the Fano resonance.

A second control is related to the detection limit. Both pathways preserve the helicity of the light, so in the cross‐polarized configurations of Figure 3e neither channel is allowed; apart from residual laser light, these spectra are featureless, the largest deviation from the background over the relevant range being 2.9σ. The cross‐polarized spectra therefore define the noise level σ under identical conditions and exclude an instrumental origin of the antiresonance. The three contributions to Equation (1) can be compared with this noise level. At 8 T in $z\left(\sigma^{+},\sigma^{+}\right)z^{\prime }$ the background is $R\approx 2.5\times 10^{4}$ counts and the noise level $\sigma \approx 2.6\times 10^{2}$ counts. With a=0.073 the bare resonant contribution $a^{2}R\approx 1.3\times 10^{2}$ counts lies at 0.5σ and is undetectable, whereas the interference term $2aR\approx 3.6\times 10^{3}$ counts is observed at 13.7σ: it is the interference, not the resonant channel alone, that renders the transition observable. Two alternatives are excluded: An incoherent sum gives $I=R\;[1+a^{2}/\left(\varepsilon^{2}+1\right)]\geq R$ everywhere and cannot dip below the background, and at $\phi =-90^{\circ }$ the dispersive term of Equation (1) vanishes, so the symmetric Faraday dip is not a derivative‐like profile. The same comparison explains the absence of a feature in the singly charged dots of Figure S12, where $R=3.0\times 10^{3}$ and σ=62 counts give a 3σ bound a<0.031, against which a=0.073 would have shown at 7.1σ. It further demonstrates that a pure trion or exciton absorption does not account for detecting the Fano AR, since an absorption line should appear in the background intensity, for both the neutral as well as singly charged QDs. Besides that, we exclude shifts in the exciton energies due to polaronic effects which become significant only in QDs with diameters of a few nanometers, the energy shifts scaling as $L^{-2}$ with the dot size L [66, 67].

## 4 Conclusion

We have demonstrated that the spin–phonon Fano resonance provides spectroscopic access to spin‐dark excitons without requiring symmetry‐lowering perturbations such as in‐plane magnetic fields or nanofabricated geometries. The quantitative extraction of the coupling parameter a and interference phase ϕ from the Fano lineshape constitutes a spectroscopic observable for the bright–dark exciton interaction that is unavailable through conventional photoluminescence. The pronounced sensitivity of the Fano resonance to the type of QD shell reveals a sign reversal of the exciton exchange energy $\delta_{0}$ at excited shells, placing the bright exciton lowest in energy—a hierarchy that may favor bright‐exciton‐mediated coherent control and could be exploited for dynamic decoupling of exchange‐coupled spins in gate operations, where precise knowledge of $\delta_{0}$ sets the pulse timing. The persistence of the effect to about 40 K and the compatibility with ensemble measurements make the technique practical for a wide range of semiconductor nanostructures. Looking ahead, extending the spin–phonon Fano spectroscopy to 2D transition metal dichalcogenides, where spin‐ and momentum‐forbidden dark excitons dominate the low‐temperature emission and couple strongly to phonons, and to perovskite nanocrystals, where the bright–dark ordering remains debated, offers a route to resolving open questions in excitonic fine‐structure physics.

## 5 Experimental Section

### 5.1 Samples

The intentionally undoped InGaAs/GaAs QDs (#14824) were grown by molecular‐beam epitaxy (MBE) on (100)‐oriented GaAs substrates. After growth, each sample was rapidly annealed at different temperatures ranging from 850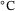 up to 970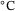. The rapid thermal annealing tunes the QD size, composition profile and, in turn, the spectral position of the emission in a precise manner. The results presented in the manuscript were obtained for the samples rapidly annealed at 870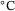 and 910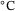. The lens shaped QDs have a maximum height of 10 nm and their diameters approach a few tens of nanometers; see atomic force microscopy image in the Supporting Information. Each sample contains 10 layers of QDs with a density of $1.4\times 10^{10}$ dots/cm^2^. An AlAs/GaAs buffer layer was grown between the QD layers and GaAs substrate as well as between the QD layers and the GaAs cap layer to reduce strain.

The singly charged QDs were realized by modulation doping with Si donors 20 nm below each QD layer. The sample, which consists of 20 layers of lens shaped QDs, was annealed at 945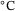 after the MBE growth.

### 5.2 Resonant Optical Excitation and Detection

The QDs were excited by the light of a tunable continuous‐wave Ti:Sapphire laser (linewidth <4μeV). Its emission was permanently controlled in energy by an interferometric wavelength meter. The laser intensity was additionally stabilized by a liquid‐crystal amplitude modulator, reducing the laser–light amplitude fluctuations to below 0.05%. The laser light was focused by a quartz lens ($f_{1}$ = 400 mm) on to the sample placed inside a cryostat with a superconducting split–coil magnet. The sample was attached strain free to a rotation holder and was exposed to external magnetic fields up to 10 T. The QD emission was collected by achromatic lenses ($f_{2}$ = 250 mm) and was analyzed by a double monochromator (total focal length of 2 m) equipped with a Peltier‐cooled single‐photon counting detector. The monochromator gratings had 1800 grooves/mm and the widths of the four monochromator slits (entrance/intermediate/intermediate/exit) were typically set to 80/100/100/80 μm yielding a spectral resolution of 2.5 μeV. The exposure time was usually one second per spectral point. The efficiency of the GaAs‐based detector is energy dependent, particularly for values reaching 1.38 eV (900 nm). To correct the intensities of the spectra from 1.38 to 1.48 eV they were weighted by the function $\zeta \left(E\right)=1.028+\left(9.183\times 10^{-4}-1.028\right)/\left\{1+\exp\left[(E-E_{0})/0.0106\;\text{eV}\right]\right\}$ with $E_{0}=1.4171$ eV. The polarization of the incident and detected light was defined each by a combination of a Glan‐Taylor prism and an achromatic quarter‐wave retardation plate. The circular polarization of light is denoted by $\sigma^{\pm }$, where the signs ± are determined by the sign of the photon angular momentum projection on the optical *z*‐axis (light *k*‐vector is parallel to *z*). The light propagation direction opposite to *z* is denoted by *z'*.

Unless specified otherwise, the measurements were done at a temperature of 6 K, and the energy of the incident laser light with typical 30 mW power (measured in front of the focusing quartz lens $f_{1}$) was tuned to the center of the *p*‐shell QD PL.

## Author Contributions

J.J.S. and K.B. performed the experiments, analyzed, and interpreted the data and contributed to the writing of the manuscript. M.M.G. provided the theoretical framework and reviewed the manuscript. C.S., S.S., A.L. and A.D.W. provided the samples. J.D. supervised the experiments, analyzed, and interpreted the data and wrote the manuscript. All authors discussed the results.

## Conflicts of Interest

The authors declare no conflicts of interest.

## Supporting information


**Supporting File**: advs78207‐sup‐0001‐SuppMat.pdf.

## Data Availability

The data that support the findings of this study are available from the corresponding author on reasonable request.
