# Supplementary material for: Optical Access to Dark Excitons Via Spin–Phonon Fano Interference
Source: Adv Sci (Weinh). 2026 Oct 14:e78207. Online ahead of print. doi: 10.1002/advs.78207 (PMC13669229; doi:10.1002/advs.78207)
Supplement: Supplementary file 1 [file ADVS-9999-e78207-s001.pdf]

Supplementary Information:

Optical access to dark excitons via spin-phonon Fano interference

Janina J. Schindler,[1] Katja Barthelmi,[1] Mikhail M. Glazov,[2] Carlo Sgroi,[3] Sven Scholz,[3] Arne Ludwig,[3] Andreas D. Wieck,[3] Jörg Debus[1,4]*

[1] Department of Physics, TU Dortmund University, 44227 Dortmund, Germany
[2] Ioffe Institute, 194021 St. Petersburg, Russia
[3] Experimental Physics VI, Faculty of Physics and Astronomy, Ruhr University Bochum, 44780 Bochum, Germany
[4] Dortmund Center for Advanced Exploration of Dynamics Across Limits Using Spectroscopy (DAEDALUS), TU Dortmund University, 44227 Dortmund, Germany

*Corresponding author: joerg.debus@tu-dortmund.de

(i) Dependence of δ_0 on the magnetic field geometry. Addressing the *p*-shell exciton yields values of δ_0 extrapolated linearly from $E_{\mathrm{Fano}}(B)$ which remain practically constant for tilting the magnetic direction with respect to the QD growth axis. For an excitation energy $E_{\mathrm{exc}} \approx 1.45$ eV, this dependence is depicted in Fig. S1 for $\theta = 0°, 15°$ and $40°$, where the exciton exchange energy is approximately given by -70 μeV.

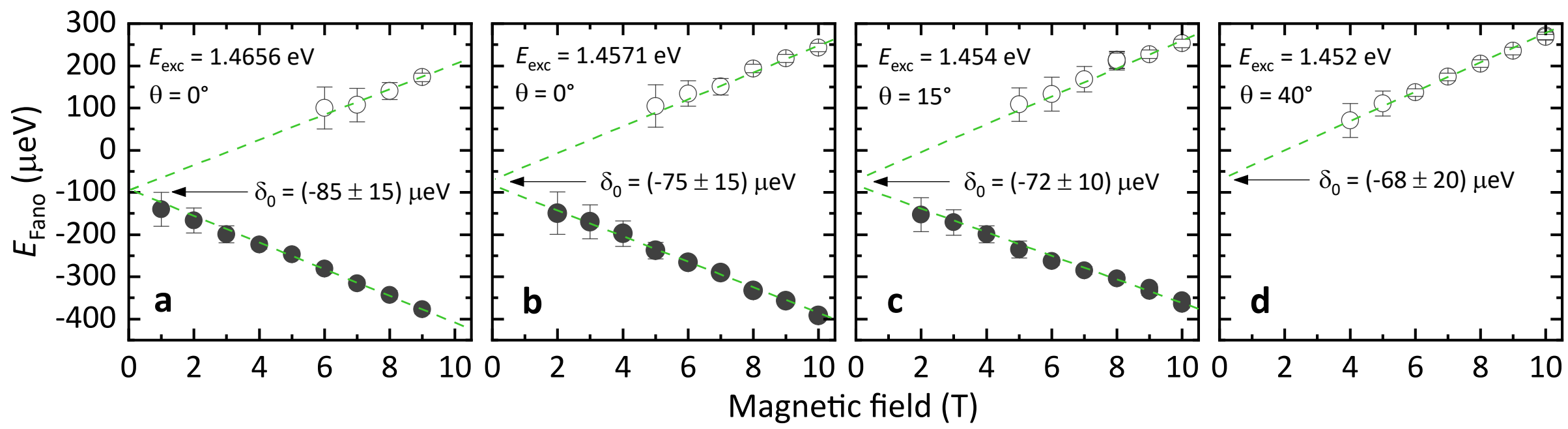

Fig. S1 | Magnetic field dependence of the spectral positions of the Fano ARs excited at **a** 1.4656 eV and **b** 1.4571 eV. The energies of the bipolar Fano resonances as function of B are shown in **c** and **d** measured at 15° and 40°, respectively. The temperature was set to 6 K, while the laser power was 30 mW.

(ii) Excitation energy dependence of the Fano resonance measured in tilted geometry at $\theta = 30° \pm 3°$. The photoluminescence of the InGaAs QD ensemble detected at 6 K under wetting-layer excitation is shown in Fig. S2a. The different QD shells are indicated. For resonantly exciting the excitons at the different shells of the QDs, the laser energy was tuned from 1.43 to 1.48 eV. The respective emission spectra in close energetic vicinity to the laser line are demonstrated as contour plots in Fig. S2b. The magnetic field strength was 6 T.

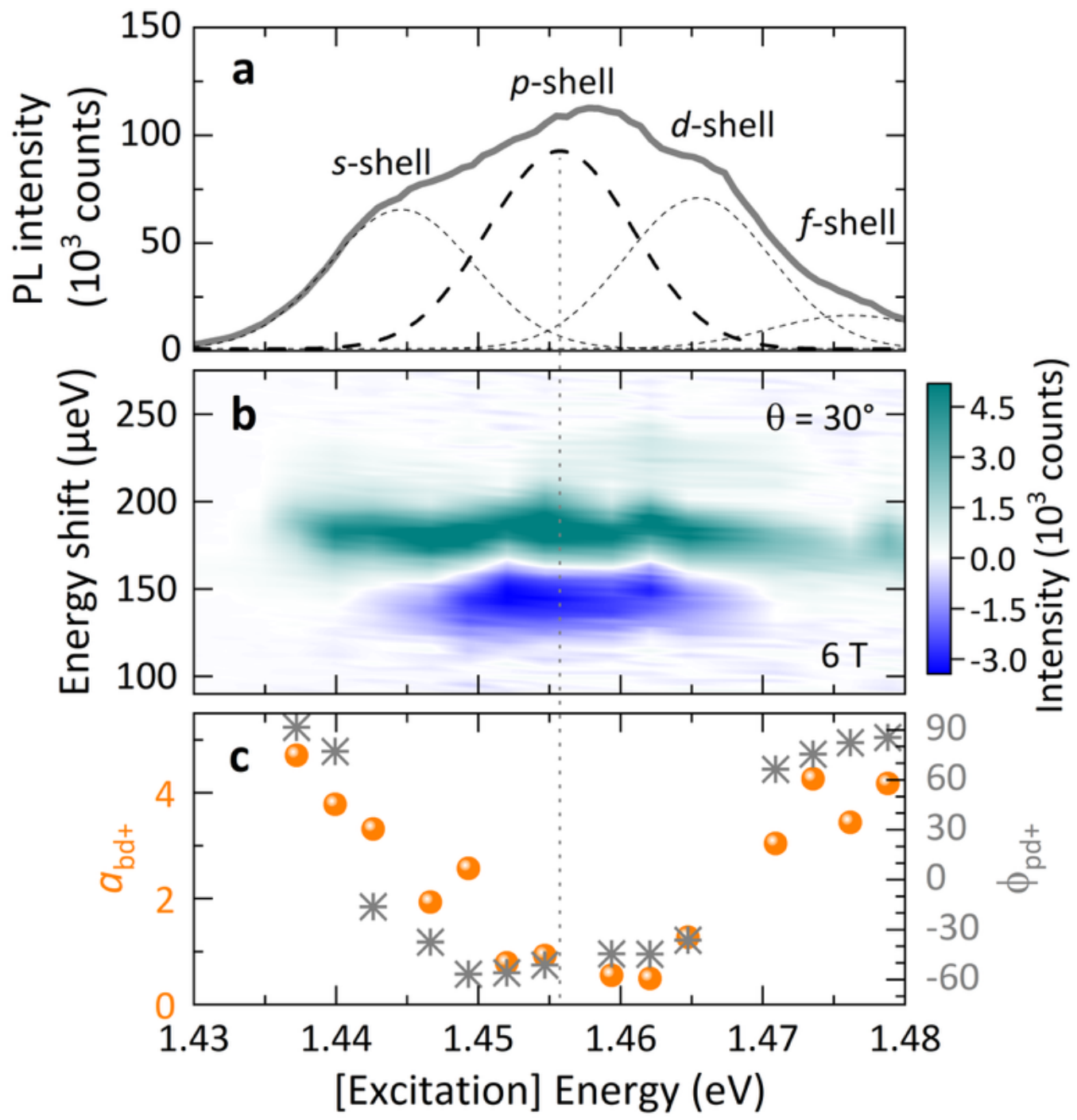

Fig. S2 | a PL of the undoped InGaAs QD ensemble. **b** Fano resonances excited across the QD ensemble in an oblique magnetic field geometry. A bipolar shape is pronounced for excitation energies ranging about the center of the *p*-shell; $T =$ 6 K, $P_{\mathrm{exc}} = 30$ mW. **c** Coupling parameter $a_{\mathrm{bd}+}$ and phase $\phi_{\mathrm{bd}+}$ of the Fano resonances involving the $|+1>$ and $|+2>$ excitons.

Particularly for resonant *p*-shell excitation, a bipolar shaped Fano resonance is observed; here, negative intensity values are given by blue color, while positive values are displayed in green. The bipolar shape is most pronounced at an excitation energy of about 1.456 eV which coincides with the center of the *p*-shell marked by the dashed vertical line. The shape becomes non-bipolar, *i.e.*, it transforms to a Lorentzian profile, for energetically leaving the *p*-shell towards the *s*-shell as well as *d*- and *f*-shell. This change in the Fano line shape is also demonstrated by the coupling parameter $a_{\mathrm{bd}+}$ and the phase $\phi_{\mathrm{bd}+}$ evaluated from fitting the Fano resonances with Eq. (1) of the main manuscript [Eq. (21), see below]; see Fig. S2c. At the *p*-shell center, $a_{\mathrm{bd}+}$ takes its smallest values of about 0.8 indicating the strongest Fano interference where resonant and continuum channels are most balanced. For excitation energies deviating from the *p*-shell, $a_{\mathrm{bd}+}$ rises to values above 3, because a is a ratio of amplitudes and therefore reports the balance of the two channels rather than the strength of either: the number of dots contributing at a given excitation energy cannot be the cause, since it multiplies both amplitudes alike and cancels from their ratio. The variation must therefore reside in $|\mu|$, Γ_{e}, and/or Γ_{b}, whose separate contributions cannot be disentangled from these data, while the absolute interference depth depends in addition on R. The phase $\phi_{\mathrm{bd}+}$ likewise varies across the shells, from about −50° at the *p*-shell center to about +85° at the *s*-shell flank and at the *d*-shell. This variation is not caused by a detuning of the scattered photon from the intermediate state $|e'\rangle$: such a detuning would also change the phase in the untilted geometry, whereas $\phi_{\mathrm{bd}+}$ remains at −90° across the whole PL band in Fig. 2g of the main manuscript. It arises instead from the mixing of the electron spin states by the tilted field, which modifies the complex ratio of the two interfering amplitudes and thereby rotates their relative phase, as described in the main text. Because the mixing angle is governed by the ratio of the off-diagonal Zeeman energy to the level splitting, and that splitting is shell dependent through δ_0, the phase then also varies with excitation energy in the tilted geometry while remaining constant in the untilted one. It is not correlated with the sign of δ_0, which is negative at the *p*-shell and at the *d*-shell alike (Fig. 2e of the main manuscript). Note that the coupling parameters and phases reported in this section were obtained in the tilted geometry θ

= 30°, whereas Fig. 2g of the main manuscript refers to the Faraday geometry θ = 0°; the two sets of values are therefore not directly comparable.

(iii) Calculation of the bright and dark exciton energies in Voigt geometry ($\theta = 90°, \mathbf{B} \parallel \mathbf{x}$)**.** The energies of the bright (b) and dark (d) excitons in the QDs (with a symmetry lower than D_{2d}) are evaluated by

$$E_{\mathrm{X},\pm1} = \frac{1}{4}\left[\mp(\delta_1 + \delta_2) + \sqrt{(2\delta_0 \mp \delta_1 \pm \delta_2)^2 + 4(g_x^{\mathrm{el}} \pm g_x^{\mathrm{hh}})^2 \mu_{\mathrm{B}}^2 B^2}\right] \text{ and}$$

$$E_{\mathrm{X},\pm2} = -\frac{1}{4}\left[\mp(\delta_1 + \delta_2) + \sqrt{(2\delta_0 \pm \delta_1 \mp \delta_2)^2 + 4(g_x^{\mathrm{el}} \mp g_x^{\mathrm{hh}})^2 \mu_{\mathrm{B}}^2 B^2}\right] \text{ [1].}$$

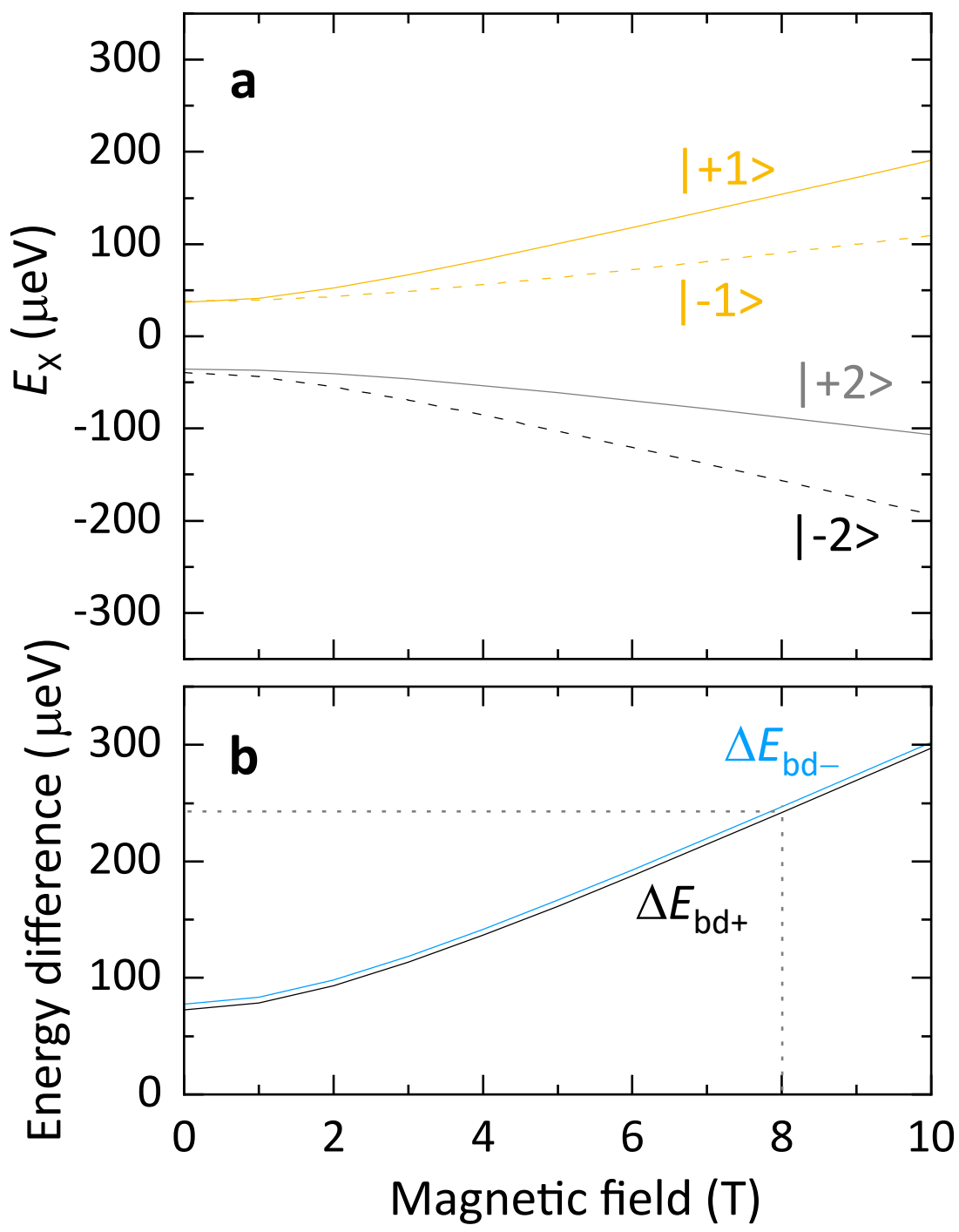

Fig. S3 | a Dark and bright exciton energies calculated for $0 \leq B \leq 10$ T in Voigt geometry. **b** Magnetic field dependence of the energy differences $\Delta E_{\mathrm{bd}+}$ and $\Delta E_{\mathrm{bd}-}$.

The exchange-based splitting between the bright and dark excitons amounts to $\delta_0 = -75$ µeV. Here δ_1 and δ_2 denote the anisotropic exchange constants that give rise to the zero-field splittings of the bright and dark doublets, respectively. The zero-field splitting of the bright (dark) exciton states reads δ_{b} = 4 µeV (δ_{d} = 1 µeV). The QD in-plane g-factors of the electron (el) and heavy hole (hh) are $g_x^{\mathrm{el}} = -0.50$ and $g_x^{\mathrm{hh}} = -0.15$ [2,3,4]. A diamagnetic shift is discarded; it would provide only weak corrections. The rotational symmetry of the QDs is disturbed by the in-plane magnetic field. It leads to a mixing of the bright and dark excitons. While in the Faraday geometry the exciton states cross each other with increasing magnetic field strength, in Voigt geometry the spin splitting demonstrates a kind of anticrossing; see Fig. S3a. In detail, the bright (dark) exciton energies are increased (reduced) by rising magnetic field. Moreover, the bright excitons lie energetically above the dark excitons due to the squaring of δ_0. Because δ_0 enters only squared, the bright-dark ordering in Voigt geometry is governed by $|\delta_0|$ alone and the sign information is lost. Determining the sign of the exchange energy therefore requires the Faraday geometry, which is the configuration used for the sign reversal reported in the main text. By taking the differences $\Delta E_{\mathrm{bd}-} = E_{\mathrm{X},-1} - E_{\mathrm{X},-2}$ and $\Delta E_{\mathrm{bd}+} = E_{\mathrm{X},+1} - E_{\mathrm{X},+2}$ we obtain the curves shown in Fig. S3b. It is obvious that these energy differences coincide with each other which contrasts with the Faraday case. Similar energy differences ($\Delta E_{\mathrm{bd}+} \approx \Delta E_{\mathrm{bd}-}$) in Voigt geometry which were probed by the Stokes and anti-Stokes spectra are also observed in experiment, cf. Ref. [5].

(iv) Gaussian-like peaks with negligible exciton exchange interaction. Gaussian-like peaks are observed in cocircular polarizations at the energy side opposite to that exhibiting the Fano resonance. At these energy shifts, the resonance condition for the bright-dark exciton spin-flip transition is not fulfilled, so only the non-resonant scattering pathway contributes and no Fano interference occurs. The Gaussian-like shape reflects the inhomogeneous broadening of the QD ensemble. Their Stokes and anti-Stokes energy shifts agree with each other and correspond to the electron Zeeman splitting $|g_z^{\mathrm{el}}B|$ with negligible excitonic exchange energies ($\delta_0 \approx 0$). These two properties are demonstrated in Fig. S4, for exciting the central part of the *p*-shell PL in oblique magnetic field geometries. Besides the magnetic field evolution of the peak positions and their linear extrapolation to 0 T (green dashed lines), in Fig. S4c the Gaussian-like peaks are presented for different magnetic fields.

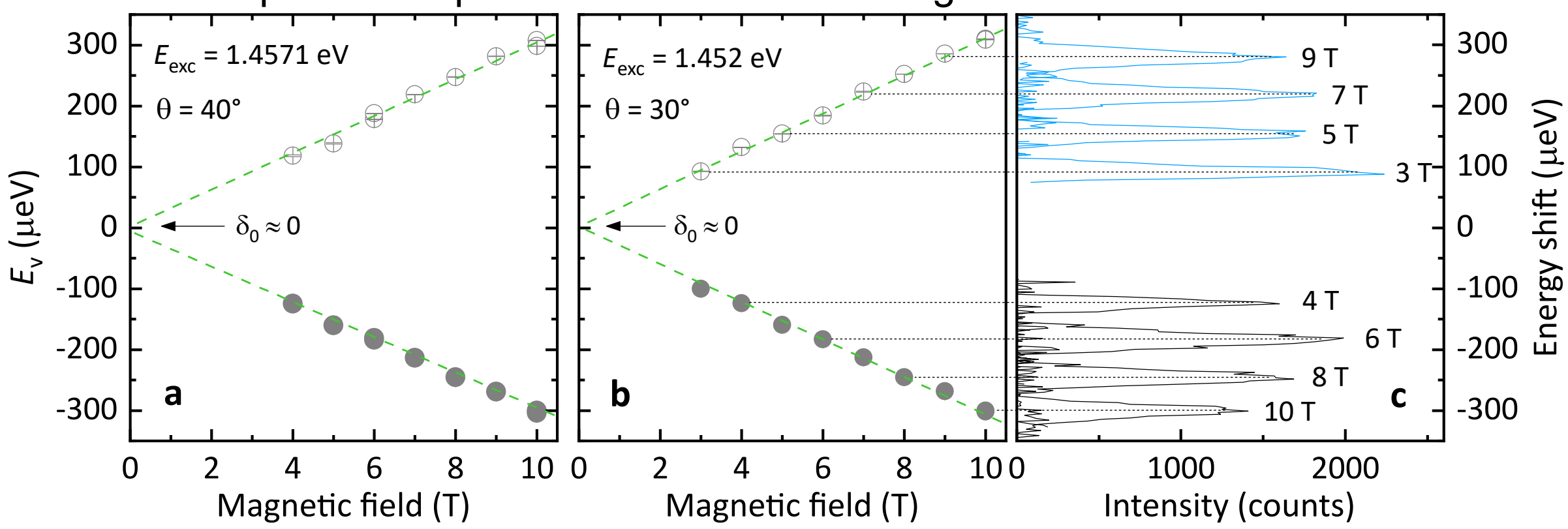

Fig. S4 | a,b Magnetic field evolution of the Gaussian-like peak positions for two different tilting angles and slightly different excitation energies. The linear extrapolations of E_v reveal negligible exciton exchange energies. **c** Spectra of the Gaussian-like peaks, for different magnetic fields, are related to the energies shown in panel b.

(v) Laser power and temperature dependences of the QD photoluminescence (PL) and the inverse coupling factor. The PL of the InGaAs QD ensemble was excited at 1.533 eV. The magnetic field (8 T) was directed along an oblique direction ($\theta = 30°$) and the temperature was 1.8 K. The dependence of the PL on the incident laser power P_{exc} is shown in Fig. S5a; the top panel contains the PL spectra (each normalized to 1) illustrated as contour plot, while the bottom panel displays exemplary PL spectra at different P_{exc}. Below 50 mW the wetting layer (WL) emission is weak, and significant changes in the PL intensity are observed among the different QD shells. While at low P_{exc} the states of mainly the lowest shell are occupied by carriers, increasing P_{exc} leads to a successive population of energetically higher lying states so that the emission from the respective shells becomes pronounced. Above 50 mW the WL PL dominates against the QD emission. The spectral position of the WL emission is identified by a photoluminescence excitation (PLE) spectrum detected at 1.456 eV (center of *p*-shell PL); see Fig. S5b. For exciting the QD ensemble at about 1.490 eV, the PL of the *p*-shell is strongly enhanced. This assignment agrees with PLE spectra obtained for other InGaAs QD ensembles [6].

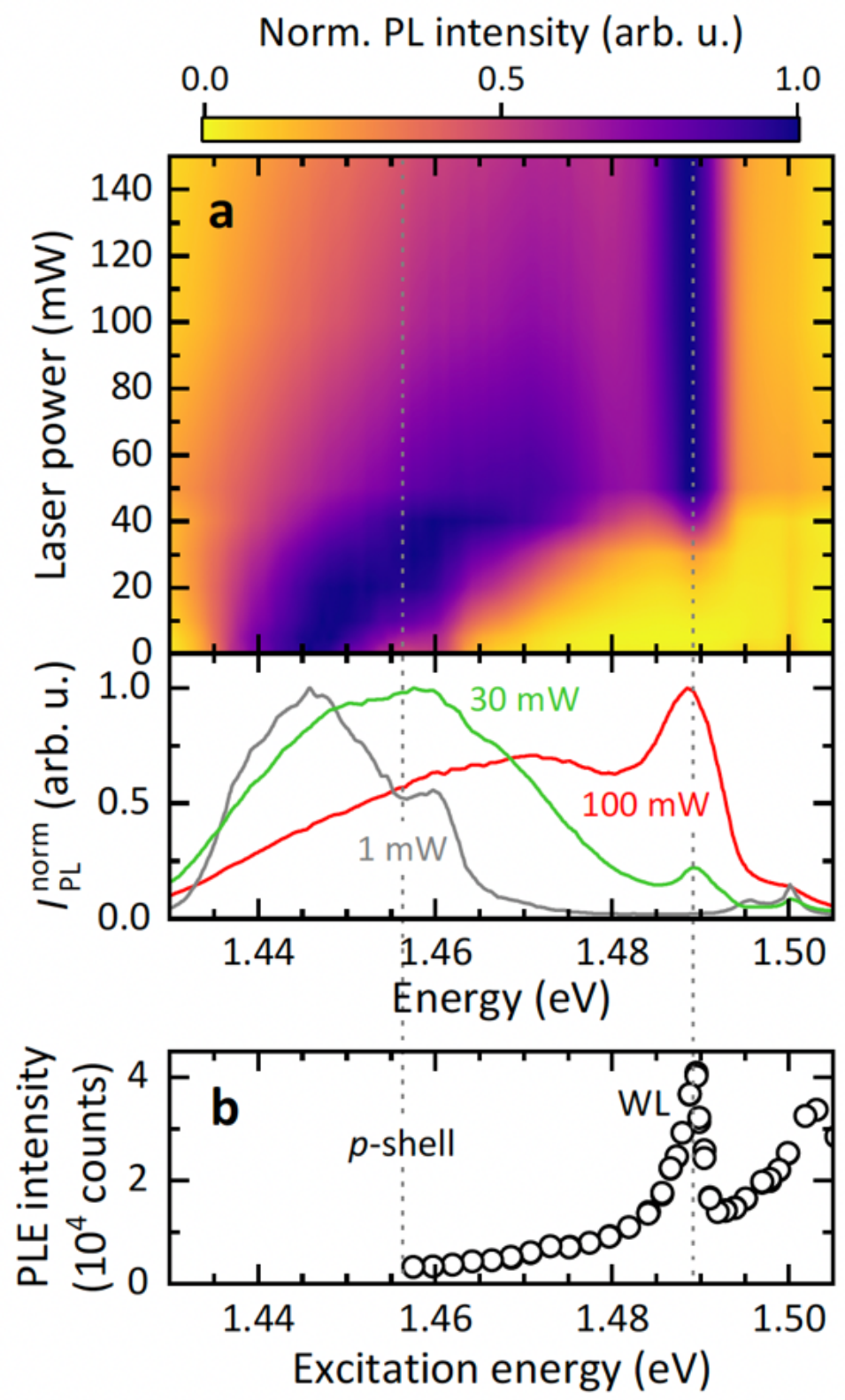

Fig. S5 | a Dependence of the QD PL on the laser power. **b** PLE measured at 6 K, 0 T and 30 mW (adjusted regularly).

The laser power dependence of the Fano resonance shape measured at $\theta = 30°$ is shown by the inverse coupling factor in Fig. S6, for the involvement of the $|+1> \rightarrow |+2>$ and $|-1> \rightarrow |-2>$ transitions in the Fano process. An increasing laser power tunes the Fano line shape from a Lorentzian profile at low P_{exc} to a bipolar shape, while large laser powers exceeding about 50 mW turn the shape back to a Lorentzian profile. Both $1/a_{\mathrm{bd}+}$ and $1/a_{\mathrm{bd}-}$ follow a similar non-monotonic dependence, peaking near 50 mW where the resonant and continuum channels are most equally balanced. This similarity confirms that the same phonon coupling mechanism governs both the Stokes and anti-Stokes Fano processes.

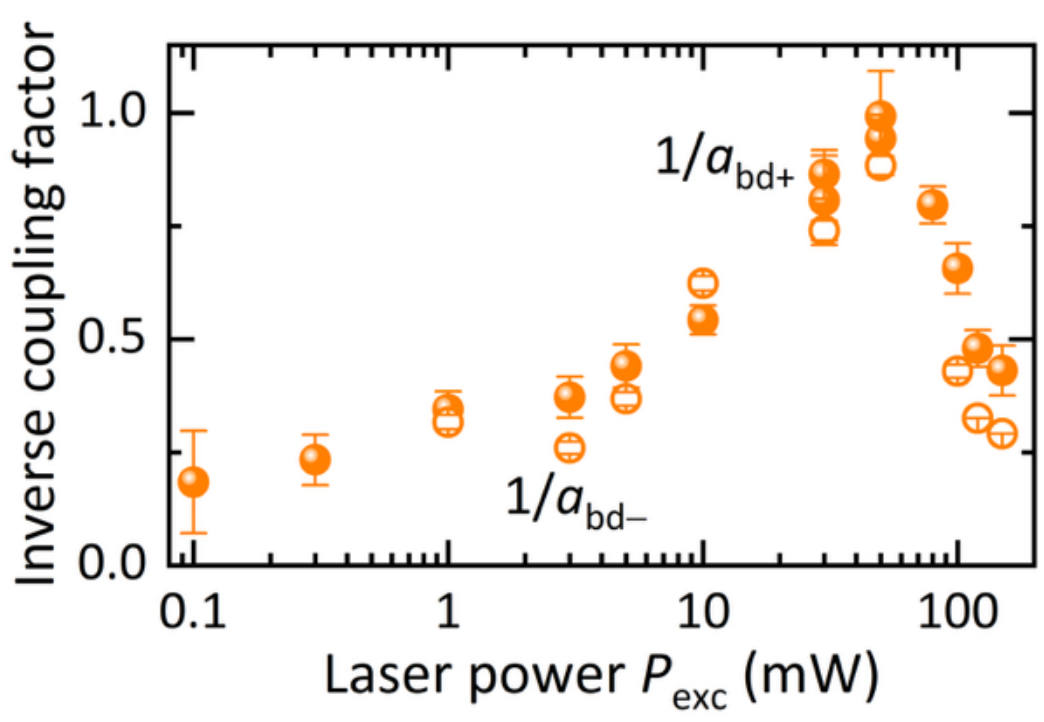

Fig. S6 | Laser power dependence of the inverse coupling factors; $T = 6$ K, $B = 8$ T.

An impact of multi-exciton interaction on the rate of the bright-dark exciton transition or, respectively, electron spin transition is instead neglected. The resonantly excited *p*-shell exciton may relax to the lower lying *s*-shell and a further *p*-shell exciton may be

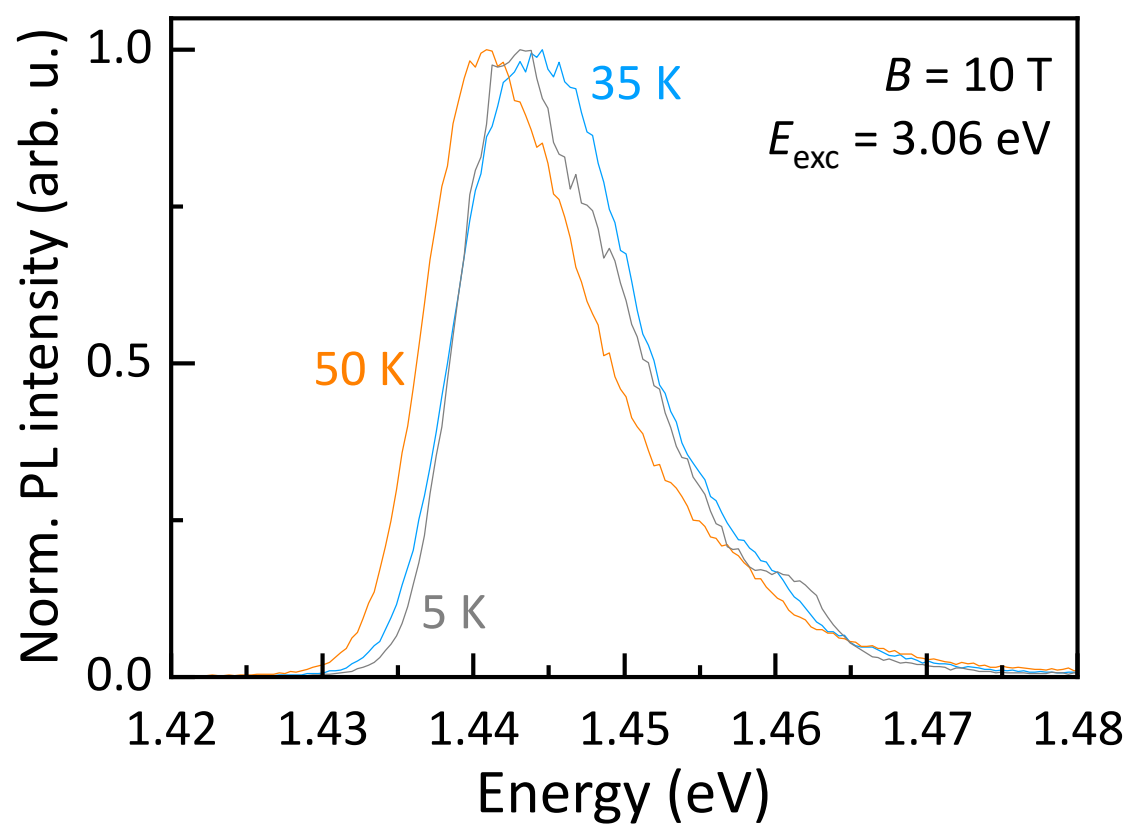

Fig. S7 | Normalized QD PL spectra measured at a non-resonant laser power of 35 mW and different temperatures.

generated optically in the same QD. The rate model of section (xii) shows that population relaxing into the *s*-shell does accumulate there, so this scenario cannot be dismissed on the grounds of the relaxation rates or of the *s*-shell exciton lifetime. It is excluded instead by the excitation-energy dependence of δ_0, which changes magnitude and sign across the PL band (Fig. 2e of the main manuscript): a process originating in a common *s*-shell reservoir would return a single value at every excitation energy, whereas the measured δ_0 tracks the *p*-shell being addressed. We do not invoke the Pauli exclusion principle to render the spin-flip rate independent of the excitation power here, since the power dependence discussed in the main manuscript is attributed to carrier-density-dependent damping.

The band gap of the InGaAs QDs is reduced with increasing temperature leading to a redshift of the emission. From 5 to 50 K the emission is shifted to low energies by about 2.8 meV, as shown in Fig. S7. This thermally induced shift is in agreement with previous measurements on singly charged InGaAs QDs [7].

(vi) LA phonon scattering spectra. Above 20 K and for resonantly probing the InGaAs QD ensemble at the *s*-shell and *p*-shell, we observe an additional line in the spectra. For $E_{\text{exc}} = 1.455$ eV, it lies at about $170\,\mu\text{eV}$; the spectral position follows the excitation energy through the Brillouin relation, although the predicted shift of about 4 μeV across the accessible range is comparable to the fit uncertainty. Moreover, it only appears in cocircular polarization configurations. In Fig. S8 two spectra measured at 0 and 8 T in longitudinal field geometry $(\mathbf{B}||\mathbf{z})$ are shown. The application of the magnetic field enhances the intensity of the LA phonon lines, both at the Stokes as well as anti-Stokes side.

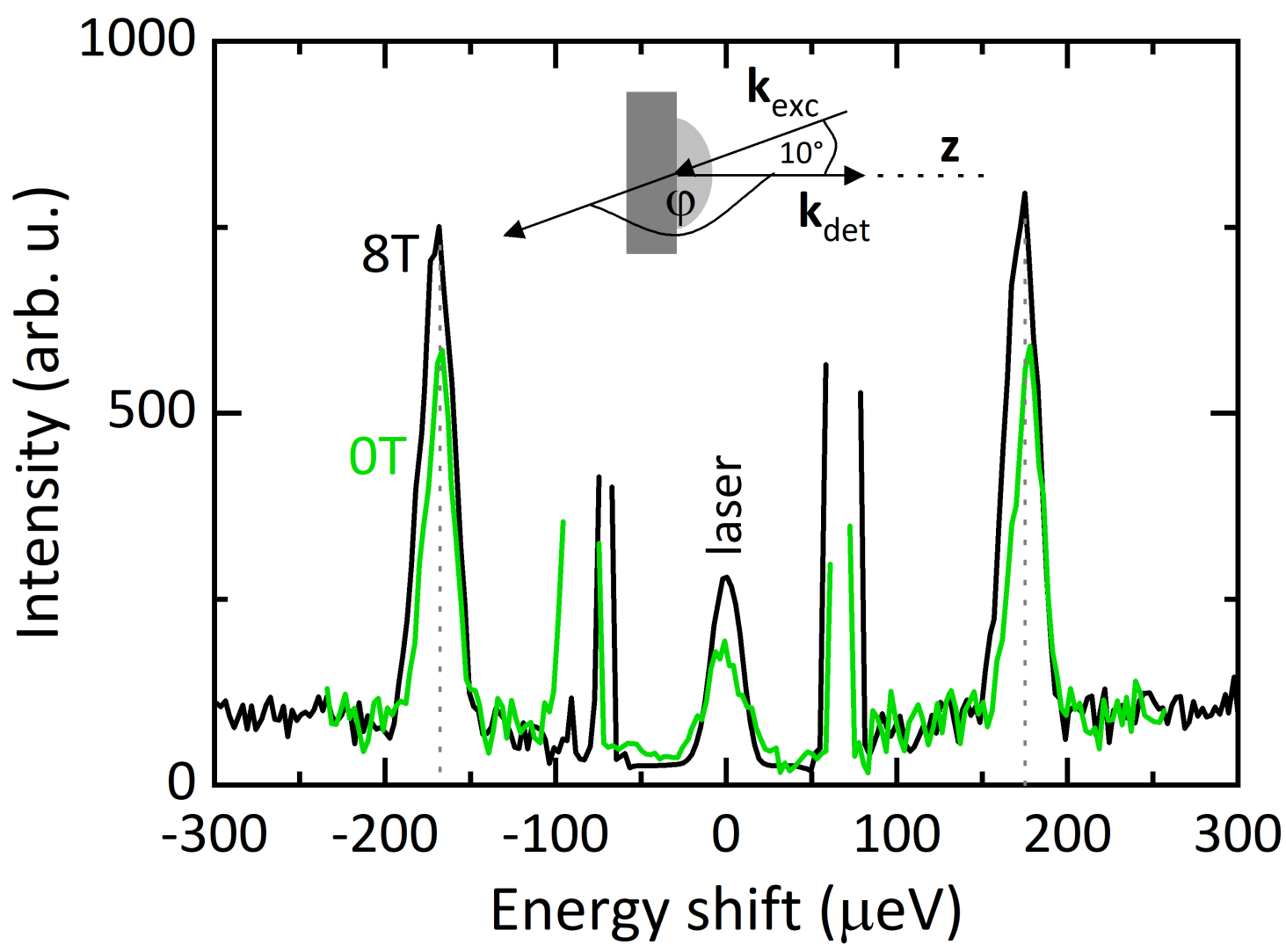

Fig. S8 | LA phonon scattering peaks at zero and 8 T external magnetic field strength; $T = 30$ K. Inset: Scheme of sample with a lens-shaped QD and the directions of the incident and detected light paths.

The spectral position follows the Brillouin-Mandelstam relation for a longitudinal acoustic phonon whose wavevector is fixed by momentum conservation in the

scattering geometry rather than by the dot dimensions: $E_{\mathrm{LA}} = 2E_{\mathrm{exc}}\left(\frac{n'}{c}\right) v_{\mathrm{L}} \sin\frac{\varphi}{2}$. The optical axes of the incident and detected light are tilted approximately 10° from each other, yielding a scattering angle $\varphi\measuredangle(\mathbf{k}_{\mathrm{exc}}, \mathbf{k}_{\mathrm{det}})$ of $170°$. The calculated energy, as described in the main text, agrees with the spectral positions of the lines detected in the experiments.

Fitting the line at 8 T gives an apparent full width at half maximum of (21.7 ± 0.4) µeV on the Stokes and (22.7 ± 0.5) µeV on the anti-Stokes side. The laser linewidth and the monochromator resolution are separate contributions, both much smaller than 22 µeV (see Experimental Section), so that after deconvolution the width is about 20 µeV, that is essentially the measured width. Any inhomogeneous contribution (spread of dot sizes and orientations, and the range of wavevectors accepted by the collection optics) can only broaden the observed line, so this figure is an upper bound on the homogeneous width and the coherence length inferred from it is correspondingly a lower bound. The corresponding phonon coherence length $\hbar v_{\mathrm{L}}/\Delta E$ is then at least 140 nm, more than three times the dot diameter of (40 ± 4) nm. Since the acoustic impedances of dot and barrier differ by less than 2%, a vibration confined to the dot would be strongly leaky and correspondingly broad, so a line this narrow is difficult to reconcile with confinement; we note that a long coherence length is evidence for, but not proof of, a spatially unconfined displacement field. The preservation of the helicity underlines that purely radial acoustic modes (l = 0) are probed, whose spherical symmetry does not disturb the polarization properties [8,9]. The intensity enhancement in magnetic field is of electronic origin: the field shifts the exciton states relative to the fixed laser energy, changing the resonant denominators of both scattering pathways and hence the scattering cross-section. No confinement of the acoustic modes by the magnetic field is implied.

(vii) Angular components of the exciton-phonon coupling and the electronic envelopes. In InGaAs/GaAs QDs the acoustic phonons are typically treated as bulk-like, since the acoustic impedance mismatch between the QD and the barrier/wetting layer material is small. The acoustic impedance is generally determined by the mass density and sound velocity: ρv. Both the mass densities ($\rho^{\mathrm{InGaAs}} = 5350$ kg/m^3 and $\rho^{\mathrm{GaAs}} = 5310$ kg/m^3) [10,11,12] as well as the longitudinal ($v_{\mathrm{L}}^{\mathrm{InGaAs}} = 4670$ m/s and $v_{\mathrm{L}}^{\mathrm{GaAs}} = 4770$ m/s) and transversal ($v_{\mathrm{T}}^{\mathrm{InGaAs}} = 3290$ m/s and $v_{\mathrm{T}}^{\mathrm{GaAs}} = 3368$ m/s) sound velocities are comparable to each other [13,14,15]. Hereby, we consider a QD composition of $In_{0.10}Ga_{0.90}As$. The simultaneous observation of l = 0 and l = 1 phonon signatures does not require the acoustic modes to be confined. Expanding the phonon plane wave in spherical harmonics (Rayleigh expansion), the exciton-phonon coupling contains all angular components, and which of them contributes is fixed by the symmetry of the initial and final exciton envelopes rather than by any quantization of the phonon spectrum. The l = 0 component connects envelopes of equal parity, whereas the l = 1 component is odd under inversion. The spin flip itself is not produced by these orbital factors: the deformation-potential and piezoelectric interactions are diagonal in spin, and the bright-dark transition is enabled by the spin-orbit admixture of opposite-parity, opposite-spin components into the confined states, as set out in the main manuscript. The odd-l orbital factor is what renders the resulting matrix element

non-vanishing. Consistent with the small impedance mismatch quoted above, the acoustic phonons are therefore treated throughout as bulk-like, with a continuous spectrum labelled by the wavevector k. The expressions given below are accordingly to be read as the angular components of the exciton-phonon coupling at a given wavevector, not as eigenmodes of a bounded elastic sphere; note that the dots imaged in section (x) are lens-shaped, with a height of about 10 nm and a diameter of (40 ± 4) nm.

No elastic boundary at the dot surface is assumed anywhere in our analysis and the phonon spectrum remains continuous throughout; the relations below serve only to define the longitudinal and transverse sound velocities used above, in terms of the mass density and the elastic constants. The acoustic-phonon displacement field (amplitude) $\boldsymbol{u}_{l,n}$ then results from Navier's equation to $\rho\,\partial^2\boldsymbol{u}_{l,n}/\partial t^2 = (\lambda + \kappa)\nabla(\nabla\boldsymbol{u}_{l,n}) + \kappa\nabla^2\boldsymbol{u}_{l,n}$ [16]. Here, l labels the angular component of the coupling and n the radial index of the corresponding Bessel-function factor; neither denotes an eigenmode of a bounded elastic sphere. The Lamé coefficients λ and κ also define the longitudinal and transversal sound velocities: $v_{\mathrm{L}} = \sqrt{(2\kappa + \lambda)/\rho}$ and $v_{\mathrm{T}} = \sqrt{\kappa/\rho}$ [17]. The displacement $\boldsymbol{u}_{l,n}$ is described by the spherical harmonics $Y_{lm}(\theta,\varphi)$ with the azimuthal quantization number $|m| \leq l$ and the spherical Bessel functions $j_l(k_n r)$ of the first kind [18,19,20]. The angular component of the coupling is thus defined by $\psi_{lm}(k_n r) = j_l(k_n r)Y_{lm}(\theta,\varphi)$. Here, the phonon wavevector is k_n, which may be expressed by ω/v_{L} or ω/v_{T}, and the position vector is r. The angular components are degenerate in m, so we set m = 0 and label the components by l alone. For l = 0 we obtain:

$$\psi_{00}(k_1 r) = j_0(k_1 r)Y_{00}(\theta,\varphi) = \sqrt{\frac{1}{4\pi}\frac{\sin(k_1 r)}{k_1 r}} \quad .$$

For l = 1 the corresponding component is:

$$\psi_{10}(k_1 r) = j_1(k_1 r)Y_{10}(\theta,\varphi) = \sqrt{\frac{3}{4\pi}}\left[\frac{\sin(k_1 r)}{(k_1 r)^2} - \frac{\cos(k_1 r)}{k_1 r}\right]\cos\theta \quad .$$

These angular components have the same functional form as the envelopes of *s*- and *p*-shell electrons confined in QDs [21,22,23]. The angular components of the coupling are non-vanishing only for those l permitted by the symmetry and parity of the electronic envelopes they connect. Their radial profiles differ: the electron envelope in these dots is not a spherical Bessel function.

The angular components of the coupling and the electron envelopes are given by $|\psi_{lm}|^2$. They are shown in Fig. S9 for l = 0 and for $l = 1$ modes which correspond to the electronic *s*- and *p*-shell, respectively. The $l = 0$ case depicted in Fig. S9a is described by a radial-symmetric distribution concentrated at the QD center, as

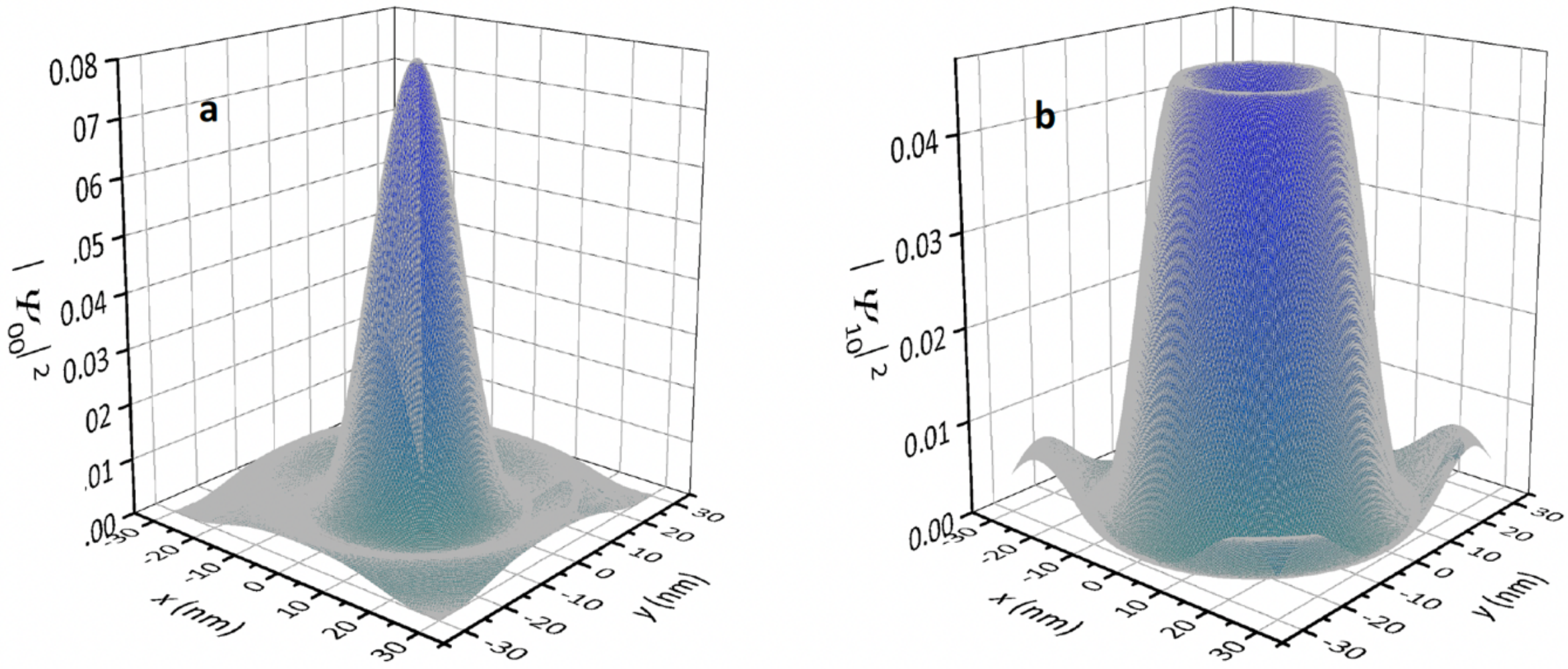

Fig. S9 | a,b Angular components of the exciton-phonon coupling form factor, ψ_{00} and ψ_{10}, for $r = \sqrt{x^2 + y^2}$ and $k_1 = 2\pi/(4 \cdot 10^{-8})$ 1/m.

characteristic of an *s*-type envelope combined with the l = 0 component of the coupling. For $l = 1$, the form factor tends to zero at the center of the *xy*-plane yielding a dipolar-like distribution; see Fig. S9b. It is assigned to *p*-shell electron envelopes and to the transverse branch of the coupling [24]. A cross-section of the radial distribution of the respective squared amplitude evaluated over a dot radius of about 20 nm is depicted in Fig. S10. It possesses a node at the center of the QD and maxima near its boundary. The transverse acoustic phonons and the electron interact with each other more strongly when the coupling form factor and the electron envelope overlap spatially.

In real QDs, different elongations in the in-plane directions and distribution alignments along the *z*-direction which depend on the composition profile as well as dot size and shape may change the widths and heights of the coupling form factors and of the electron envelopes. Thus, they may be differently strong localized within the QDs.

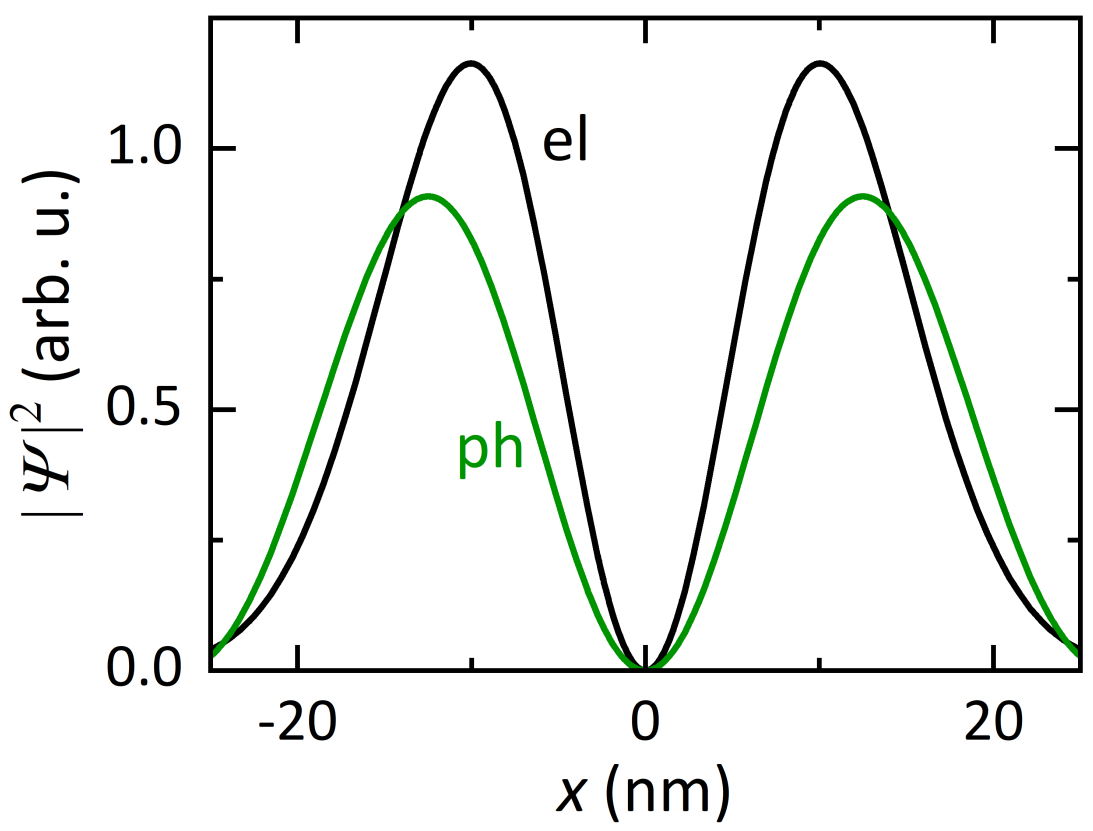

Fig. S10 | Cross-section ($y = 0$ nm) of the radial distribution of the squared l = 1 coupling form factor and the *p*-shell electron envelope, for a dot radius of about 20 nm.

(viii) Fano resonance spectra measured in transmission geometry. In transmission geometry with $\mathbf{k}_{\text{exc}}||\mathbf{k}_{\text{det}}$ and a slightly oblique magnetic field orientation ($\theta = 15°$), the cocircularly polarized Fano resonances have the same bipolar shape as in the reflection geometry; see Fig. S11. The parameters a and ϕ of the Fano processes involving the $|+1> \rightarrow |+2>$ and $|-1> \rightarrow |-2>$ transitions are consistent between reflection and transmission geometries, and remain unchanged upon reversal of the magnetic field polarity. In the transmission geometry, an on-axis excitation with $\mathbf{k}_{\text{exc}}||\mathbf{k}_{\text{det}}$ is performed. Due to the tilting of the sample by $\theta = 15°$, the polarization vector of the laser light however acquires a component parallel to the sample surface normal [25], causing a weak cross-polarization leakage: a nominally σ^- polarized excitation partially addresses the $|+1>$ exciton state, and vice versa. In this cross-polarized channel, the leakage intensity is too weak to produce a bipolar Fano resonance; only a Gaussian-shaped line is detected.

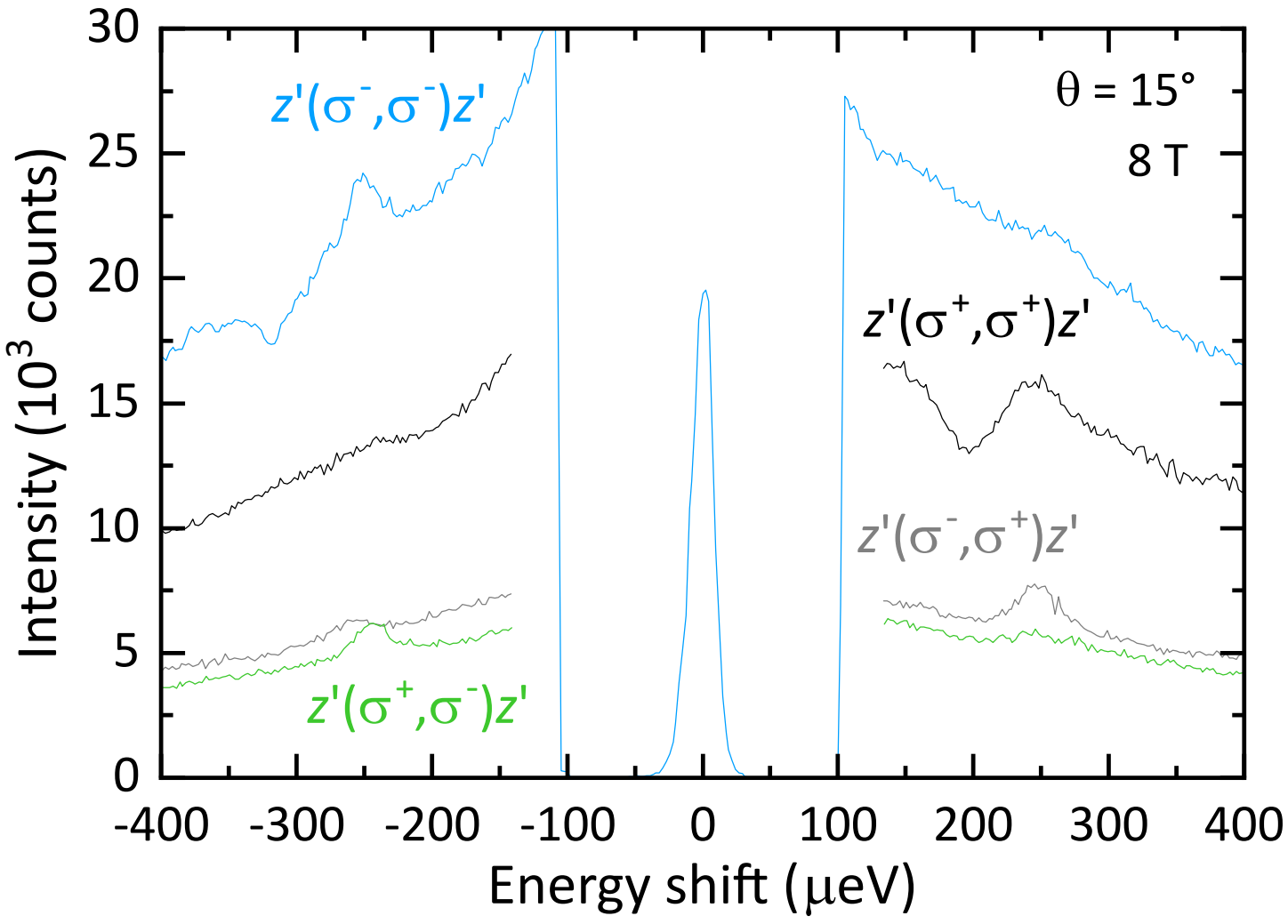

Fig. S11 | For resonantly exciting the *p*-shell of the undoped InGaAs QDs in transmission geometry, Fano resonances are detected in the cocircularly polarized configurations; $T = 6$ K, $E_{\text{exc}} = 1.465$ eV, $P_{\text{exc}} = 30$ mW.

(ix) Resonant excitation of singly charged InGaAs QDs at the *p*-shell. The QDs are charged in average by a single electron [5]. Neither a Fano AR in Faraday geometry (Fig. S12a) nor a bipolar shaped Fano resonance in a tilted geometry (Fig. S12b) are observed. Instead, a Gaussian shaped line is detected in tilted geometry only whose energy shift corresponds to the electron Zeeman splitting [5,6]. At 30° and 6 T, the electron Zeeman splitting is given by 187 μeV. Due to the resident electrons in these dots, merely negative trions are excited; dark excitons cannot be probed. The line in the spectrum at the Stokes or anti-Stokes side is caused by the exchange interaction between the electrons of the trion; the line itself thus

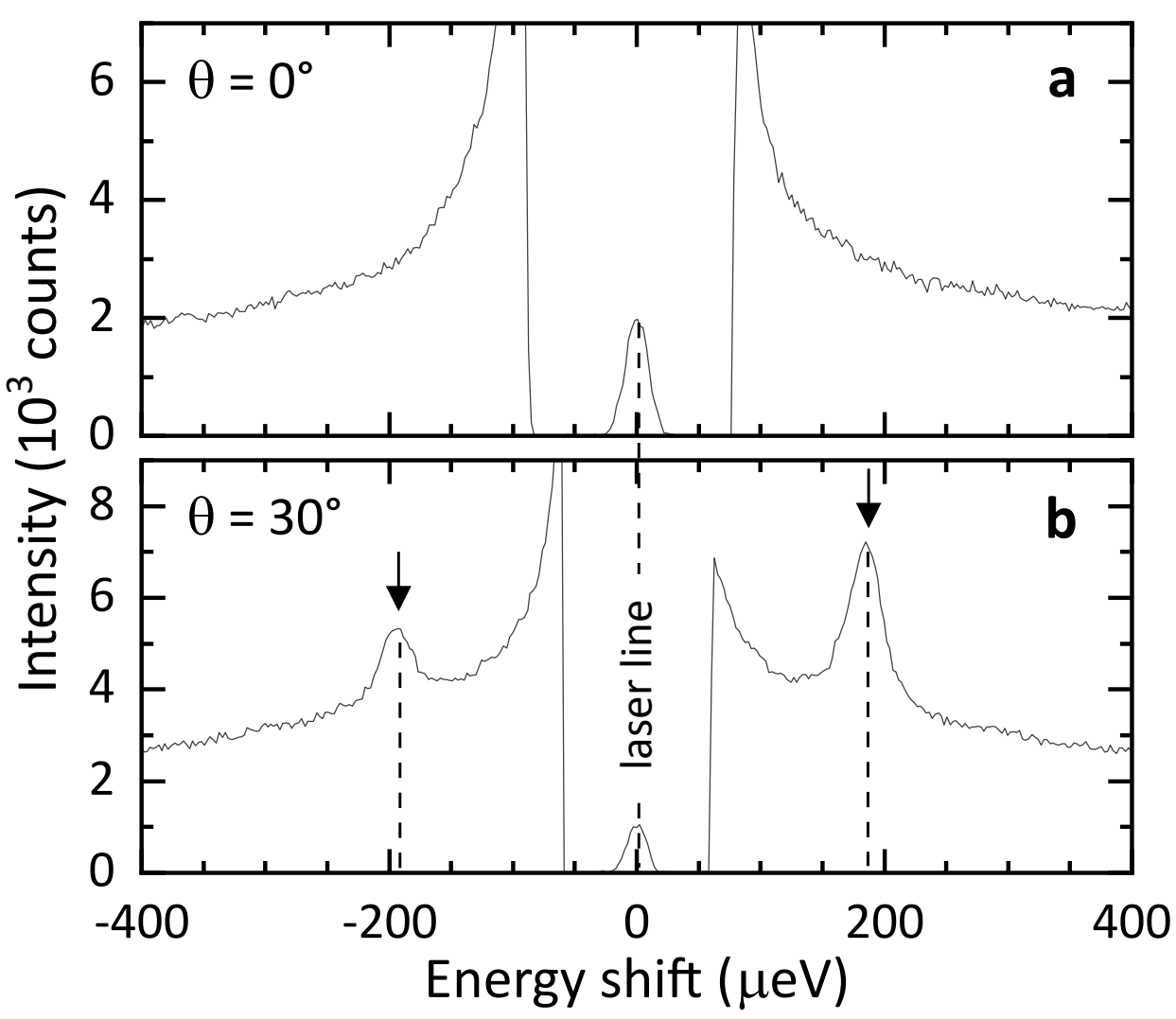

Fig. S12 | Emission spectra close to the laser line for exciting negative trions at the QD *p*-shell emission; $B = 6$ T, $T = 6$ K, $E_{\text{exc}} = 1.448$ eV, $P_{\text{exc}} = 30$ mW. **a** In Faraday geometry only a background emission is observed. **b** In tilted geometry ($\theta = 30°$) a sharp emission line is detected at the Stokes and anti-Stokes side.

indicates the flip of an electron spin accompanied by an energy change equal to the electron Zeeman splitting.

(x) Atomic force microscopy (AFM) image of the InGaAs QD ensemble. A top view on a $3 \times 3\ \mu m^2$ area of InGaAs QDs is shown in Fig. S13. They were imaged by atomic force microscopy prior to the capping process. The spatial resolution of the atomic force microscope was better than 0.1 nm. The height of the QD ensemble shown lies in average slightly above 10 nm. The average diameter of the dots is measured to (40 ± 4) nm.

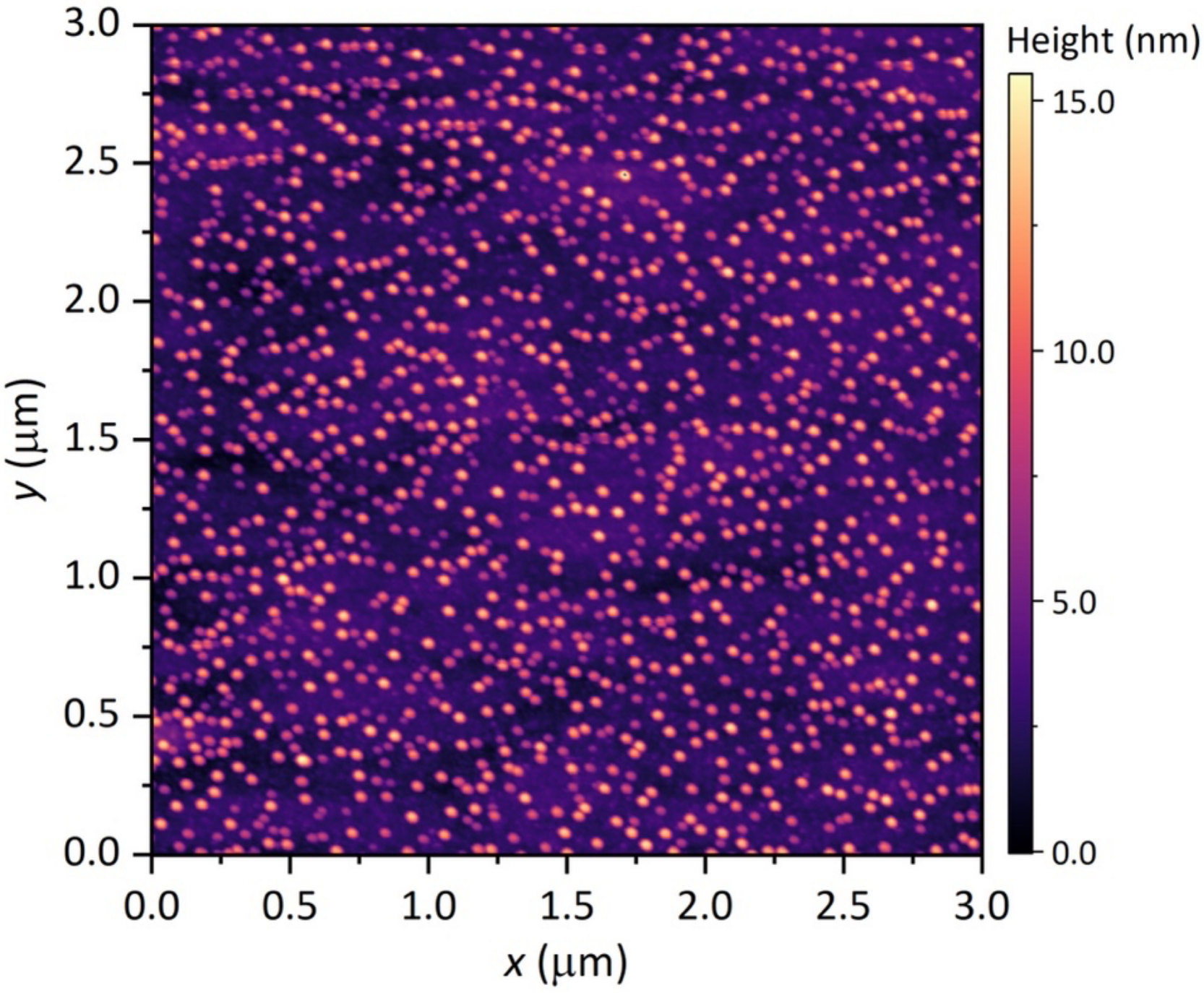

Fig. S13 | AFM image of an ensemble of InGaAs/GaAs QDs.

(xi) Theoretical framework of the spin-phonon Fano resonance effect.

I. Generic model of light scattering

a. Preliminaries

Let us consider two electronic states $|\downarrow\rangle$ and $|\uparrow\rangle$ and let Δ_Z be the spin splitting between these states. For definiteness, let $|\downarrow\rangle$ be the ground state of the spin system, *i.e.*, $\Delta_Z >$ 0. Under the continuous-wave resonant excitation used here the populations are maintained by the optical pumping cycle rather than by thermal equilibrium, so no assumption of thermal depopulation of the upper spin state is required; in the experiment $k_B T$ in fact exceeds Δ_Z. The treatment applies to either sign of Δ_Z, the roles of phonon emission and absorption being interchanged when the dark state lies above the bright one, as is the case for the (−) Zeeman branch. We denote $|\mathrm{e}\rangle$ and $|\mathrm{e}'\rangle$ the excited states of the electronic system coupled to the states $|\downarrow\rangle$ and $|\uparrow\rangle$ via the electromagnetic field. Further, we extend the treatment to account for excitons as initial and final states.

The standard *spin-flip Raman scattering* process can be described as an electron transition

$$|\downarrow\rangle \otimes |\hbar\omega_\mathrm{i}, \sigma_\mathrm{i}\rangle \to |\mathrm{e}\rangle \to |\uparrow\rangle \otimes |\hbar\omega_\mathrm{f}, \sigma_\mathrm{f}\rangle \ , \quad (1)$$

where $\omega_\mathrm{i}, \sigma_\mathrm{i}$ and $\omega_\mathrm{f}, \sigma_\mathrm{f}$ are the frequency and polarization state of, respectively, the incident (i) and scattered (f) photon. The composite matrix element for the process (1) takes the form

$$M_{\rm sf} = \frac{M^{\sigma_{\rm f}}_{\uparrow,\rm e} M^{\sigma_{\rm i}}_{\rm e,\downarrow}}{\hbar\omega_{\rm i} - E_{\rm e} + i\Gamma_{\rm e}} , \quad (2)$$

where $M^{\sigma_{\rm f}}_{\uparrow,\rm e}$ and $M^{\sigma_{\rm i}}_{\rm e,\downarrow}$ are the corresponding electron-photon interaction matrix elements, $\Gamma_{\rm e}$ is the excited state damping in the units of energy, and we set the origin of the energy at $E_{\downarrow} \equiv 0$. Generally, one needs to sum over all possible intermediate states e in the system.

The scattered light intensity takes the form

$$I_{\rm sf} \propto \frac{2\pi}{\hbar} |M_{\rm sf}|^2 \delta(\hbar\omega_{\rm i} - \hbar\omega_{\rm f} - \Delta_{\rm Z}) . \quad (3)$$

The δ-function in Eq. (3) describes the energy conservation law. It is broadened because of two effects:

1. Finite lifetime of excited $|\uparrow\rangle$ state, *i.e.*, due to the spin relaxation processes that lead to transitions $|\uparrow\rangle \rightarrow |\downarrow\rangle$; at a non-zero temperature there are also transitions $|\downarrow\rangle \rightarrow |\uparrow\rangle$.
2. Inhomogeneous broadening in the system due to spread of g-factors and, correspondingly, of the Zeeman splittings $\Delta_{\rm Z}$ in a real system.

Using the diagram technique [26,27] one can show that in Eq. (3) the δ-function is replaced by

$$\delta(x) \rightarrow \Delta(x) = \int_{-\infty}^{\infty} d\Delta_{\rm Z} \mathcal{F}(\Delta_{\rm Z}) \frac{1}{\pi} \frac{\Gamma_{\uparrow}}{(x-\Delta_{\rm Z})^2 + \Gamma_{\uparrow}^2} , \quad (4)$$

where $\Gamma_{\uparrow}$ is the damping of the $|\uparrow\rangle$ spin state, $\mathcal{F}(\Delta_{\rm Z})$ is the distribution function of Zeeman splittings. It corresponds to the inclusion of electron-phonon interaction in the Greens functions ($\uparrow\downarrow$). In what follows, we disregard the inhomogeneous broadening for brevity. As a result, for the scattered intensity we have

$$I_{\rm sf} \propto \frac{2}{\hbar} |M_{\rm sf}|^2 \frac{\Gamma_{\uparrow}}{(\hbar\omega_{\rm i} - \hbar\omega_{\rm f} - \Delta_{\rm Z})^2 + \Gamma_{\uparrow}^2} . \quad (5)$$

Equation (5) describes the "standard" profile of the spin-flip Raman scattering.

The *phonon-assisted light scattering* includes the electron-phonon scattering in the excited state:

$$|\downarrow\rangle \otimes |\hbar\omega_{\rm i}, \sigma_{\rm i}\rangle \rightarrow |\rm e\rangle \rightarrow |\rm e'\rangle \otimes |\hbar\Omega_{\bf k}\rangle \rightarrow |\downarrow\rangle \otimes |\hbar\Omega_{\bf k}\rangle\, |\hbar\omega_{\rm f}, \sigma_{\rm f}\rangle , \quad (6)$$

where $|\hbar\Omega_{\bf k}\rangle$ is the state of a phonon with the wavevector $\mathbf{k}$. It is described by third-order composite matrix element

$$M_{\rm ph} = \frac{M^{\sigma_{\rm f}}_{\downarrow,\rm e'} V^{\bf k}_{\rm e'e} M^{\sigma_{\rm i}}_{\rm e,\downarrow}}{(\hbar\omega_{\rm i} - E_{\rm e} + i\Gamma_{\rm e})(\hbar\omega_{\rm i} - E_{\rm e'} - \hbar\Omega_{\bf k} + i\Gamma_{\rm e'})} . \quad (7)$$

The scattered light intensity is given by

$$I_{\rm ph} \propto \frac{2\pi}{\hbar} \sum_{\bf k} |M_{\rm ph}|^2\, \delta(\hbar\omega_{\rm i} - \hbar\omega_{\rm f} - \hbar\Omega_{\bf k}) . \quad (8)$$

Generally, the summation over all intermediate states e and e′ is assumed. This process can be considered (in the case of acoustic phonon scattering) as Brillouin-Mandelstam scattering. For real intermediate states, where $\hbar\omega_{\rm i} \approx E_{\rm e}$ and $\hbar\omega_{\rm f} \approx E_{\rm e'}$, Eqs. (7) and (8) describe the photoluminescence of the system, cf. Refs. [27–29]. It can be seen by summing in Eq. (8) over $\mathbf{k}$ using the energy-conservation δ-function. The

Lorentzian factors from the matrix element provide the frequency dependence of the absorption and emission, respectively.
Naturally, the photon emission can involve $|\uparrow\rangle$ spin state as a final state. This process is described by

$$|\downarrow\rangle \otimes |\hbar\omega_{\rm i}, \sigma_{\rm i}\rangle \rightarrow |{\rm e}\rangle \rightarrow |{\rm e}'\rangle \otimes |\hbar\Omega_{\mathbf{k}}\rangle \rightarrow |\uparrow\rangle \otimes |\hbar\Omega_{\mathbf{k}}\rangle\, |\hbar\omega_{\rm f}, \sigma_{\rm f}\rangle \ . \quad (9)$$

The corresponding matrix element for such a spin-flip phonon-assisted scattering is given by

$$M_{\rm ph,sf} = \frac{M_{\uparrow,{\rm e}'}^{\sigma_{\rm f}}\, V_{{\rm e}'{\rm e}}^{\mathbf{k}}\, M_{{\rm e},\downarrow}^{\sigma_{\rm i}}}{(\hbar\omega_{\rm i} - E_{\rm e} + i\Gamma_{\rm e})(\hbar\omega_{\rm i} - E_{{\rm e}'} - \hbar\Omega_{\mathbf{k}} + i\Gamma_{{\rm e}'})} \ . \quad (10)$$

b. Spin-flip ↑→↓ broadening of δ-function in Eq. (3) and derivation of Eq. (5)

Let us consider in more detail the generalized light scattering process. Naturally, the “final” state of the electronic system in the studied process, $|\uparrow\rangle$, is not the ground state of the system. Hence, it can decay to the ground state, $|\downarrow\rangle$, emitting photons. Accordingly, in an actual scattering process the final state of the system is

$$|\downarrow\rangle \otimes |\hbar\omega_{\rm f}\rangle \otimes |\hbar\Omega_{\mathbf{k}}\rangle \ , \quad (11)$$

namely, the electron in the ground state, photon and phonon, rather than $|\downarrow\rangle \otimes |\hbar\omega_{\rm f}\rangle$ (without phonon).
Neglecting the damping of the photon and phonon states, we have instead of Eq. (2) for the scattering matrix element

$$\widetilde{M}_{\rm sf} = \frac{V_{\downarrow\uparrow}^{\mathbf{k}}\, M_{\uparrow,{\rm e}}^{\sigma_{\rm f}}\, M_{{\rm e},\downarrow}^{\sigma_{\rm i}}}{(\hbar\omega_{\rm i} - E_{\rm e} + i\Gamma_{\rm e})(\hbar\omega_{\rm i} - \hbar\omega_{\rm f} - \Delta_{\rm Z} + i\Gamma_{\uparrow})} = M_{\rm sf}\frac{V_{\downarrow\uparrow}^{\mathbf{k}}}{(\hbar\omega_{\rm i} - \hbar\omega_{\rm f} - \Delta_{\rm Z} + i\Gamma_{\uparrow})} \ , \quad (12)$$

with

$$\Gamma_{\uparrow} = \pi \sum\nolimits_{\mathbf{k}} |V_{\uparrow\downarrow}^{\mathbf{k}}|^2\, \delta(\Delta_{\rm Z} - \hbar\Omega_{\mathbf{k}}) \ . \quad (13)$$

Note that if there are several relaxation pathways to the ground state, *e.g.*, via different phonon modes, the damping $\Gamma_{\uparrow}$ contains contributions from all the relaxation channels.
The scattered light intensity is given by

$$I_{\rm sf} \propto \frac{2\pi}{\hbar}\sum_{\mathbf{k}} \left|\widetilde{M}_{\rm sf}\right|^2 \delta(\hbar\omega_{\rm i} - \hbar\omega_{\rm f} - \hbar\Omega_{\mathbf{k}}) = \frac{2\pi}{\hbar}\sum_{\mathbf{k}} \frac{|M_{\rm sf}|^2}{(\hbar\omega_{\rm i} - \hbar\omega_{\rm f} - \Delta_{\rm Z})^2 + \Gamma_{\uparrow}^2} \times$$
$$\times \left|V_{\downarrow\uparrow}^{\mathbf{k}}\right|^2 \delta(\hbar\omega_{\rm i} - \hbar\omega_{\rm f} - \hbar\Omega_{\mathbf{k}}) = \frac{2}{\hbar}|M_{\rm sf}|^2 \frac{\Gamma_{\uparrow}}{(\hbar\omega_{\rm i} - \hbar\omega_{\rm f} - \Delta_{\rm Z})^2 + \Gamma_{\uparrow}^2} \quad (14)$$

in agreement with Eq. (5).

II. Interference effects in spin-flip Raman scattering and Fano-like scattered lineshape

In quantum mechanics different pathways of a process interfere if they share same initial and final states. In our case, the interference is possible if the same phonon mode is involved in both in the spin-flip process and in the Brillouin-Mandelstam light scattering. These processes and their diagrammatic representations are depicted in Figs. S14a and S14b; compare excitonic energy-level schemes in Figs. 1c and 1d of the main manuscript.

The matrix elements of the processes are given by

$$M_{\mathrm{r}} = \frac{V^{\mathbf{k}}_{\downarrow\uparrow}\, M^{\sigma_{\mathrm{f}}}_{\uparrow,\mathrm{e}}\, M^{\sigma_{\mathrm{i}}}_{\mathrm{e},\downarrow}}{(\hbar\omega_{\mathrm{i}} - E_{\mathrm{e}} + i\Gamma_{\mathrm{e}})(\hbar\omega_{\mathrm{i}} - \hbar\omega_{\mathrm{f}} - \Delta_{\mathrm{Z}} + i\Gamma_{\uparrow})} \ , \tag{15a}$$

$$M_{\mathrm{nr}} = \frac{M^{\sigma_{\mathrm{f}}}_{\downarrow,\mathrm{e}'}\, V^{\mathbf{k}}_{\mathrm{e}'\mathrm{e}}\, M^{\sigma_{\mathrm{i}}}_{\mathrm{e},\downarrow}}{(\hbar\omega_{\mathrm{i}} - E_{\mathrm{e}} + i\Gamma_{\mathrm{e}})(\hbar\omega_{\mathrm{i}} - E_{\mathrm{e}'} - \hbar\Omega_{\mathbf{k}} + i\Gamma_{\mathrm{e}'})} \ . \tag{15b}$$

In both cases, we start from a photon $\hbar\omega_{\mathrm{i}}$ and the ground state electron $|\downarrow\rangle$, and end up with the photon $\hbar\omega_{\mathrm{f}}$ and the phonon $\mathbf{k}$. In general, the summation over e and e' is needed, but we disregard it for simplicity. The intensity of the scattered light takes the form

$$I \propto \frac{2\pi}{\hbar} \sum_{\mathbf{k}} |M_{\mathrm{r}} + M_{\mathrm{nr}}|^2\, \delta(\hbar\omega_{\mathrm{i}} - \hbar\omega_{\mathrm{f}} - \hbar\Omega_{\mathbf{k}}) \ . \tag{16}$$

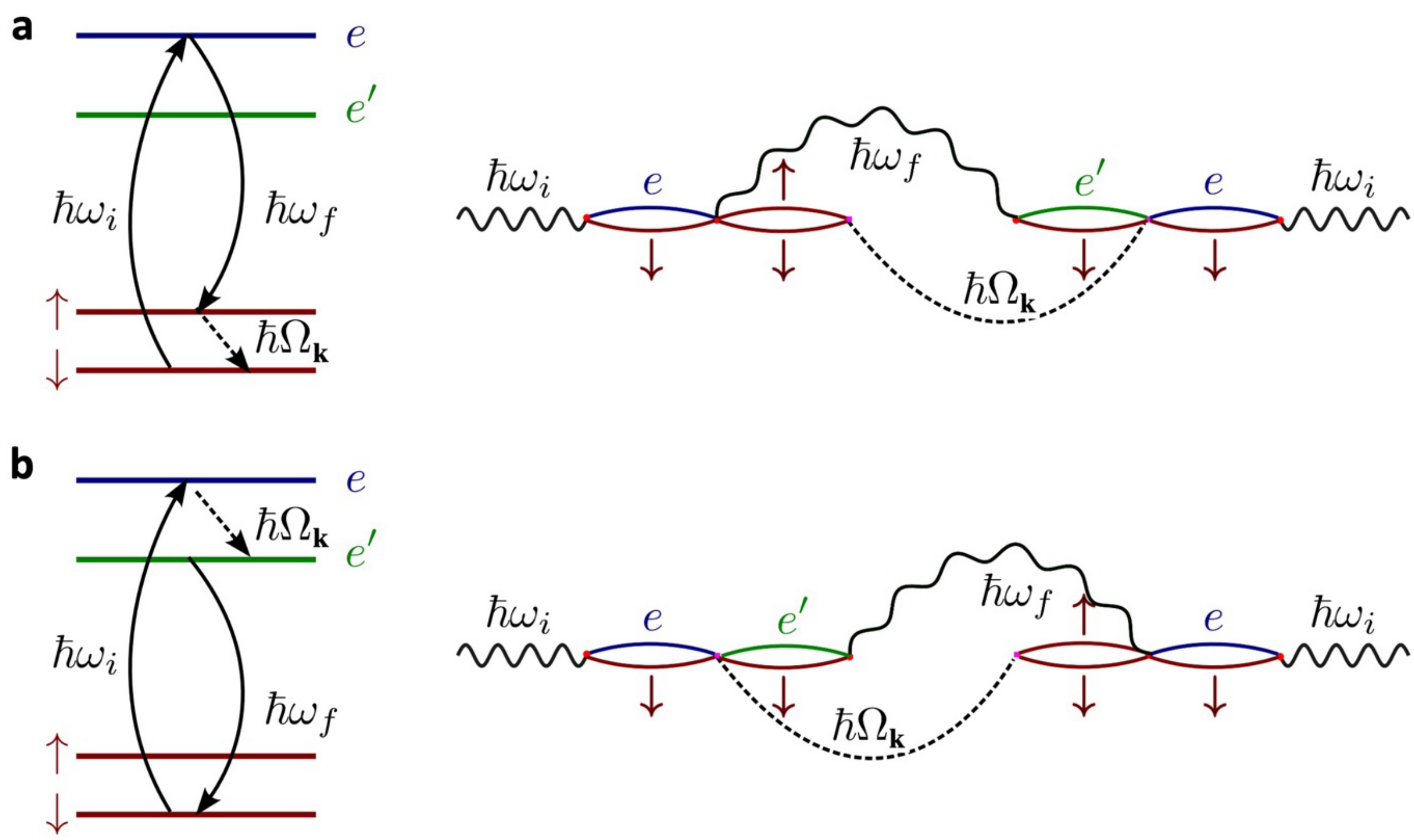

Fig. S14 | Energy-level scheme and diagrammatic representation of **a** the resonant and **b** the non-resonant scattering process.

We introduce the following notations [cf. Eq. (13)]:

$$\Gamma_{\mathrm{s}} = \pi \sum_{\mathbf{k}} V^{\mathbf{k}}_{\mathrm{ee}'} V^{\mathbf{k}}_{\downarrow\uparrow}\, \delta(\hbar\omega_{\mathrm{i}} - \hbar\omega_{\mathrm{f}} - \hbar\Omega_{\mathbf{k}}) \ , \tag{17a}$$

$$\tilde{\Gamma}_{\mathrm{e}'} = \pi \sum_{\mathbf{k}} \left|V^{\mathbf{k}}_{\mathrm{ee}'}\right|^2 \delta(\hbar\omega_{\mathrm{i}} - \hbar\omega_{\mathrm{f}} - \hbar\Omega_{\mathbf{k}}) \ , \tag{17b}$$

$$\varepsilon = \frac{\hbar\omega_{\mathrm{i}} - \hbar\omega_{\mathrm{f}} - \Delta_{\mathrm{Z}}}{\Gamma_{\uparrow}} \ . \tag{17c}$$

Note that Γ_{s} is, generally, complex. These quantities together with $\Gamma_{\uparrow}$ smoothly depend on the reduced energy ε. We also introduce

$$\mu = \frac{M^{\sigma_{\mathrm{f}}}_{\uparrow,\mathrm{e}}}{M^{\sigma_{\mathrm{f}}}_{\downarrow,\mathrm{e}'}} \ , \quad D = \hbar\omega_{\mathrm{i}} - E_{\mathrm{e}} + i\Gamma_{\mathrm{e}} \ , \quad D' = \hbar\omega_{\mathrm{f}} - E_{\mathrm{e}'} + i\Gamma_{\mathrm{e}'} \ , \tag{17d}$$

that are also complex, in general.
We obtain from Eqs. (15) and (16) for the Raman intensity

$$I \propto R(\omega_{\rm i},\omega_{\rm f}) \times \left\{1 + |\mu|^2 \frac{|D'|^2}{\Gamma_\uparrow \tilde{\Gamma}_{e'}} \frac{1}{\varepsilon^2+1} + 2{\rm Re}\left[\mu \frac{D'\Gamma_{\rm s}}{\Gamma_\uparrow \tilde{\Gamma}_{e'}} \frac{\varepsilon - i}{\varepsilon^2+1}\right]\right\} \quad , \tag{18}$$

where

$$R(\omega_{\rm i},\omega_{\rm f}) = \frac{2}{\hbar} \frac{\left|M^{\sigma_{\rm f}}_{\downarrow,{\rm e}'} M^{\sigma_{\rm i}}_{{\rm e},\downarrow}\right|^2}{|DD'|^2} \quad , \tag{19}$$

is a smooth function of the incident and scattered frequencies that provides a background. In the QD ensemble this background is not constant across the spectral range of a Fano resonance: it is the non-resonant Brillouin-Mandelstam continuum, whose weight follows the inhomogeneous distribution of the resonantly excited dots. In fitting the measured profiles we therefore represent R by a Gaussian envelope centered close to the Fano energy. Its fitted width of about 22 μeV is well above the instrumental resolution and is therefore a real feature of the background. We treat this envelope as an empirical description of the falling weight of the continuum away from the center of the resonantly excited sub-ensemble; we do not assign its width to a specific mechanism, since the Brillouin shift itself varies by only about 4 μeV across the whole excitation-energy range. The envelope is used solely to describe the falling weight of the continuum away from the center of that distribution and carries no resonant structure of its own. Holding the coupling parameter and the phase at their quoted values, this reproduces the bipolar profiles of Fig. 3a at θ = 30°.
Let us recast

$$\mu \frac{D'\Gamma_{\rm s}}{\Gamma_\uparrow \tilde{\Gamma}_{\rm e}} = \frac{|\mu D'\Gamma_{\rm s}|}{\Gamma_\uparrow \tilde{\Gamma}_{e'}} \exp(i\phi) \quad ,$$

where the phase ϕ smoothly depends on the frequencies. Hence, from Eq. (18) we have

$$I \propto R(\omega_{\rm i},\omega_{\rm f}) \times \left\{1 + \frac{1}{\varepsilon^2+1}\left[\frac{|\mu D'|^2}{\Gamma_\uparrow \tilde{\Gamma}_{e'}} + 2\frac{|\mu D'\Gamma_{\rm s}|}{\Gamma_\uparrow \tilde{\Gamma}_{e'}}(\varepsilon\cos\phi + \sin\phi)\right]\right\} \ . \tag{20}$$

Equation (20) describes an asymmetric lineshape related to an interference of the spin-flip Raman scattering and phonon-assisted Brillouin-Mandelstam scattering. One can bring this expression to the Fano-like making several approximations:

1. We assume that $|\hbar\omega_{\rm f} - E_{{\rm e}'}| \ll \Gamma_{{\rm e}'}$, *i.e.*, we consider the resonant absorption regime; hence, $D' \approx i\Gamma_{{\rm e}'}$.
2. We assume that $V^{\mathbf{k}}_{{\rm ee}'}, V^{\mathbf{k}}_{\downarrow\uparrow}$ do not depend on the direction of $\mathbf{k}$, and, moreover, $\Gamma_{{\rm e}'} \approx \tilde{\Gamma}_{{\rm e}'}$, meaning that the phonon mode under study provides the main scattering contribution to the excited state e'. Thus, $|D'| \approx \tilde{\Gamma}_{{\rm e}'}$ and $|\,\Gamma_{\rm s}/\tilde{\Gamma}_\uparrow\,| \approx (\Gamma_{{\rm e}'}/\Gamma_\uparrow)^{1/2}$.

As a result,

$$\frac{|\mu D'|^2}{\Gamma_\uparrow \tilde{\Gamma}_{e'}} = |\mu|^2 \frac{\Gamma_{e'}}{\Gamma_\uparrow} = a^2 \ \ , \ \ \frac{|\mu D'\Gamma_{\rm s}|}{\Gamma_\uparrow \tilde{\Gamma}_{e'}} = |\mu|\sqrt{\frac{\Gamma_{e'}}{\Gamma_\uparrow}} = a \ ,$$

and Eq. (20) takes the form

$$I \propto R(\omega_{\rm i},\omega_{\rm f}) \times \left[1 + \frac{a^2 + 2a\,(\varepsilon\cos\phi + \sin\phi)}{\varepsilon^2+1}\right] \ . \tag{21}$$

Depending on the value of ϕ one obtains a resonance ($\cos\phi \approx 0$, $\sin\phi \approx 1$) or antiresonance ($\cos\phi \approx 0$, $\sin\phi \approx -1$) as well as all possible asymmetric

modifications, see Fig. S15. A key difference with Ref. [30] is the absence of zero in the intensity in general.

Two alternative descriptions of the measured profiles can be excluded without recourse to fit statistics. An incoherent superposition of the two channels would give $I \propto R\left[1+\frac{a^2}{\varepsilon^2+1}\right] \geq R$ at every energy, so it cannot produce an intensity below the background, whereas the antiresonances measured in Faraday geometry fall below it. A purely dispersive or derivative-like profile is excluded as well: at ϕ = −90° the term $2a\varepsilon\cos\phi\,/(\varepsilon^2+1)$ of Eq. (21) vanishes identically, and the observed lineshape is a symmetric dip. The coherent form of Eq. (21) is therefore required by the data themselves, and not merely preferred by the quality of the fit.

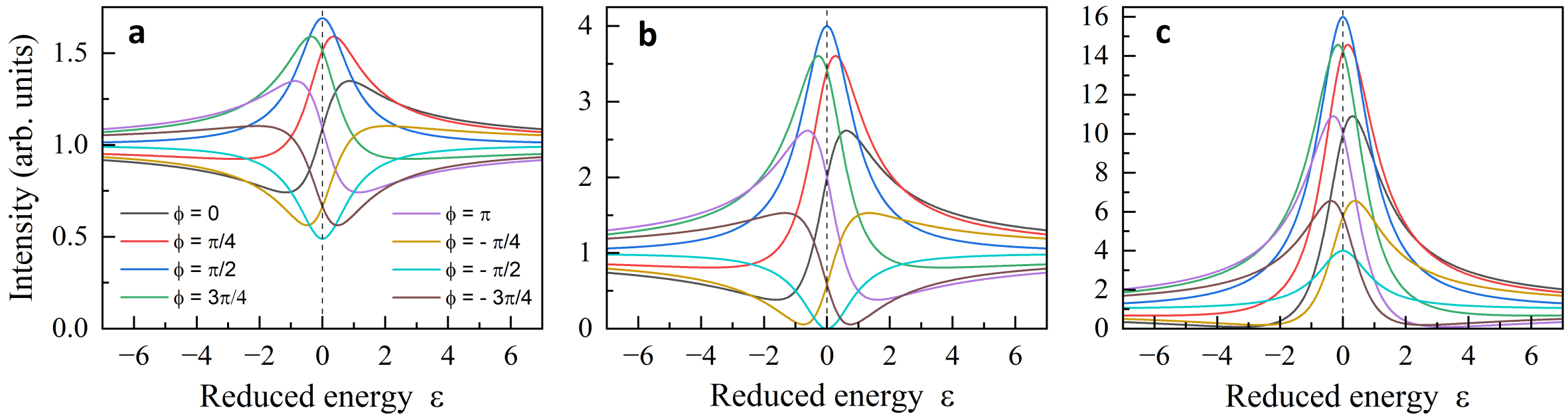

Fig. S15 | Scattering profiles calculated after Eq. (21) for different ϕ (indicated in the legends) and **a** a = 0.3, **b** a = 1, and **c** a = 3.

(xii) Steady-state dark-exciton populations under continuous-wave excitation.

The Fano process requires the dark exciton to be populated in steady state. We estimate that population with a minimal rate model for the two *p*-shell spin states, bright |+1⟩ and dark |+2⟩, of which only the bright state is fed at rate G by the resonant excitation, the dark state being populated from it by phonon-mediated electron spin flips, and drained into the *s*-shell by spin-conserving orbital relaxation at rate γ_{orb}. Detailed balance ties the two spin-flip rates through the phonon occupation n at the resonant energy, so that the downward rate carries a factor (n+1) and the upward rate a factor n.

In the steady state the pump rate, the radiative rate and the absolute magnitudes of the individual rates cancel, and the population ratio depends on a single dimensionless parameter $\rho=\tau_{\text{sf}}^{0}/\tau_{\text{orb}}$, the ratio of the spontaneous spin-flip time to the orbital relaxation time. For the (+) Zeeman branch, where the bright exciton lies above the dark one at 8 T, $\frac{N_{\text{d}}}{N_{\text{b}}}=(n+1)/(n+\rho)$; for the (−) branch, where the ordering is reversed, $\frac{N_{\text{d}}}{N_{\text{b}}}=n/(n+1+\rho)$. Over the plausible range ρ = 2 – 10^5 the ratio rises monotonically with temperature, by a factor of seven to nine between 6 and 60 K for $\rho \geq 10^2$, while remaining below unity (Fig. S16a). The dark-state occupation is therefore not depleted thermally; the loss of the Fano feature at elevated temperature cannot be attributed to a loss of dark excitons.

Two consequences are worth noting. First, the two Zeeman branches carry intrinsically different dark populations: at 6 K the ratio of the (+) to the (−) branch values is 2.9, set by detailed balance and the two phonon occupations and nearly independent of ρ over

the plausible range (Fig. S16b). This contributes to the observed Stokes/anti-Stokes intensity asymmetry, which is examined quantitatively below. Second, the model does not by itself identify the *p*-shell dark exciton as the initial state: population relaxing into the *s*-shell accumulates there, and for any plausible parameter set the *s*-shell dark reservoir is three to four orders of magnitude more occupied than the *p*-shell dark state at low temperature, falling to a factor of about 500 at 60 K (Fig. S16d). The assignment of the Fano process to the *p*-shell rests on the measured exchange energy, δ_0 = −75 μeV as against +45 μeV at the *s*-shell, and on the excitation-energy dependence of its sign (Fig. 2e of the main manuscript), not on populations.

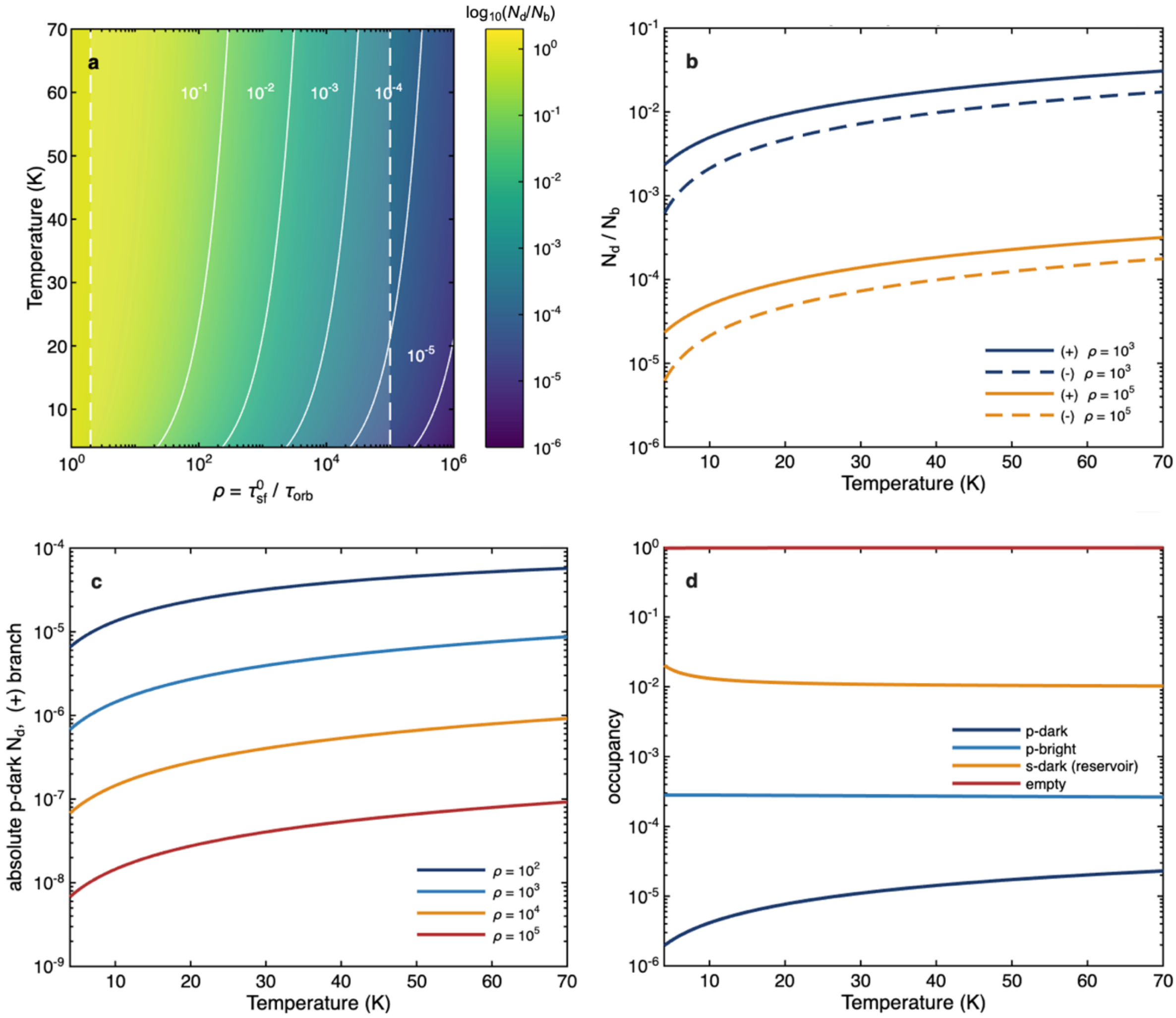

Fig. S16 | Steady-state dark-exciton populations under continuous-wave resonant excitation, evaluated at the measured bright-dark splittings at 8 T, $\Delta E_{\mathrm{bd+}}$ = 193 μeV and $|\Delta E_{\mathrm{bd-}}|$ = 332 μeV. a Ratio $N_{\mathrm{d}}/N_{\mathrm{b}}$ for the (+) Zeeman branch as a function of the rate ratio $\rho = \tau_{\mathrm{sf}}^0/\tau_{\mathrm{orb}}$ and temperature; white lines are contours of $\log_{10}(N_{\mathrm{d}}/N_{\mathrm{b}})$, and the dashed lines mark the plausible range ρ = 2 to 10^5, corresponding to τ_{orb} = 10 – 50 ps with τ_{sf}^0 = 100 ps – 1 μs. **b** The two Zeeman branches compared at ρ = 10^3 and 10^5; the (−) branch lies lower because its dark state is the upper one and must be populated by phonon absorption. **c** Absolute dark population of the (+) branch for ρ = 10^2 to 10^5, which unlike the ratio depends on the excitation and drain rates and is shown assuming these are temperature independent. **d** Occupancies of the five-state *p*/*s* shell model at ρ = 333. The *s*-shell dark reservoir exceeds the *p*-shell dark population by a factor of about 6000 at 6 K and 500 at 60 K, so the assignment of the Fano process to the *p*-shell rests on the measured exchange energy rather than on populations.

Comparison with experiment bears the first of these out. The antiresonance carries the interference term of Eq. (21), whose amplitude is $2aR$; because the profile is symmetric ($\phi \approx -90°$) the antisymmetric term $2a\varepsilon\cos\phi$ vanishes identically, so the lineshape determines the product $2aR$ and the phase but not a and R individually. Fitting the spectra of Fig. 2c separately for the two circular polarizations gives a Stokes/anti-

Stokes amplitude ratio of 0.73, 0.96, 1.40, 1.24 and 1.30 at 6, 7, 8, 9 and 10 T, a rise by a factor of about 2. The Stokes antiresonance can be located at 5 T but its amplitude is no longer extracted reliably below 6 T; this is a detection limit rather than a physical suppression: $\Delta E_{\mathrm{bd}+}$ falls to 118 μeV at 6 T and to 87, 55 and 24 μeV at 5, 4 and 3 T, so the feature approaches both the fitted linewidth Γ_{b}, which lies between 13 and 18 μeV over this range, and the spectral region
masked by the laser line, and can no longer be separated from it. The anti-Stokes branch, which disperses to larger energy shifts, remains resolved across the whole field range.
The branch asymmetry expected from detailed balance, $[N(\Delta E_{\mathrm{bd}+})+1]/N(|\Delta E_{\mathrm{bd}-}|)]$, is 2.9 and varies by less than 3 % across this interval; it is the same quantity as the population ratio quoted above rather than a second, independent constraint, since in the large-ρ limit the two coincide. Being field independent, it cannot generate the observed field dependence, and the measurement lies a constant factor of about three below it. A difference in the detection efficiency for σ+ and σ− light cannot be the origin of the field dependence either: such a difference is a property of the collection optics and is therefore independent of magnetic field and temperature, so it can contribute only a constant factor and not an evolution with either. Consistent with this, reversing the polarity of the magnetic field interchanges the two Zeeman branches while leaving every optical component untouched, and the coupling parameters and phases are unchanged under this reversal in the transmission geometry in which the test was carried out.
The same comparison can be made as a function of temperature. Extracting the peak-to-peak amplitude of the two branches from the 8 T data of Fig. 4c between 6 and 35 K, the range over which the bipolar profile is present, and dividing by the detailed-balance prediction at the corresponding resonance energies gives a quotient of 0.30 ± 0.11, to be compared with 0.39 ± 0.10 obtained from the field series above. The scatter is appreciably larger here, because at θ = 30° the profile is bipolar and its amplitude is determined less precisely than the depth of a symmetric antiresonance, so this is a consistency check rather than a precision measurement. Within that limitation the two quotients agree: the departure of the branch asymmetry from detailed balance is one and the same constant factor in the field and in the temperature series, which is what a branch-dependent coupling and a polarization-dependent detection efficiency would produce.
We note that the dark-state population and the phonon Bose factor enter the scattered intensity as a product, not as alternatives, together with the spectral density, the matrix elements and the detection efficiency. Since the branch-dependent couplings are not known independently, no parameter-free prediction of the absolute ratio is possible. What the data establish is the more restricted statement that neither the population ratio nor the Bose factors, both field independent, can account for the observed field dependence.
We emphasize what the rate model does and does not establish. It computes populations only (Fig. S16a–d). The Fano lineshape is a coherence problem, and the shape parameters a and ϕ are not obtained from these rate equations. Within the linear-response, unsaturated treatment of Eq. (21), however, both interfering amplitudes originate from the same dark exciton, so N_{d} enters only as a common intensity prefactor and cancels from a and ϕ, which are ratios of amplitudes. The

measured temperature dependence of a therefore cannot be a population effect, whereas the background intensity, which does scale with N_d, remains a legitimate population question (Fig. S16c). The model further assumes that G, γ_orb and ρ are temperature independent, that the two Zeeman branches are dynamically decoupled, and that bright and dark states share the same orbital drain.

(xiii) Temperature dependence of the LA phonon scattering line. The line at about 170 μeV described in section (vi) can be followed as a function of temperature at fixed magnetic field, using the data of Fig. 4c of the main manuscript. It must be read in the two configurations in which the Fano resonance does not overlap it: the anti-Stokes side of *z*(σ+,σ+)*z*′ and the Stokes side of *z*(σ−,σ−)*z*′. In the remaining two configurations the Fano feature lies at +193 μeV and −332 μeV at 8 T and contaminates the line below about 40 K.

Fitting a Gaussian on a linear background in these two channels gives a line at |*E*| = 172 μeV with a full width at half maximum of 22.2 μeV for $T \geq 45$ K, reproducing the widths quoted in section (vi). The line rises above the noise floor at 25 K and grows monotonically thereafter. Its integrated intensity follows the thermal occupation of the acoustic phonon mode: on the anti-Stokes side the area is proportional to $N(E_\mathrm{LA}, T)$ with a coefficient of determination $R^2 = 0.92$, and on the Stokes side to $N(E_\mathrm{LA}, T) + 1$ with $R^2 = 0.87$ (Fig. S17a).

Because the Stokes and anti-Stokes features here correspond to the same transition, their intensity ratio provides a detailed-balance test, which is not the case for the two Fano branches: those are governed by different Zeeman-split pairs rather than by phonon emission and absorption on one transition. The measured ratio is 0.99 at 65 K and 1.02 at 75 K (Fig. S17b). The larger values obtained at 45 and 55 K, 1.55 and 1.31, are consistent with residual Fano weight on the Stokes side at those temperatures. The feature is therefore identified unambiguously as one-phonon inelastic scattering by a thermally populated acoustic mode.

The comparison with the Fano resonance is instructive. Over the same interval in which the acoustic phonon occupation at E_LA grows by a factor of about four and the LA line grows with it, the bipolar contrast of the Fano resonance is lost (Fig. 4c,d of the main manuscript). The disappearance of the Fano feature at elevated temperature therefore cannot be attributed to a shortage of acoustic phonons of the appropriate energy, in agreement with the argument given in the main text and with the spectral densities of Fig. 1f.

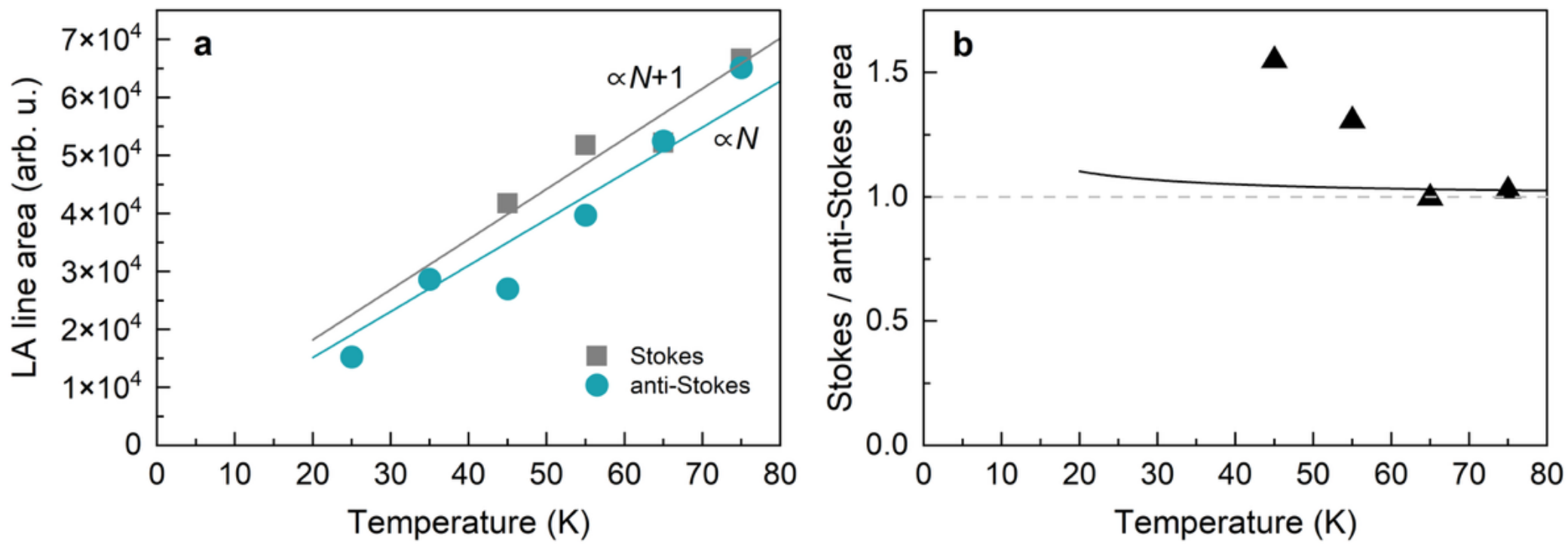

Fig. S17 | Temperature dependence of the LA phonon scattering line at 8 T. a Integrated area of the line against temperature for the anti-Stokes side of *z*(σ+,σ+)*z*′ (circles) and the Stokes side of *z*(σ−,σ−)*z*′ (squares); curves are the phonon occupation *N* and *N*+1 scaled by a single factor each. **b** Ratio of the Stokes to the anti-Stokes area compared with the detailed-balance prediction $\exp(E_{\mathrm{LA}}/k_{\mathrm{B}}T)$ shown by the curve.

References

[1] Bayer, M., Ortner, G., Stern, O., Kuther, A., Gorbunov, A. A., Forchel, A., Hawrylak, P., Fafard, S., Hinzer, K., Reinecke, T. L., Walck, S. N., Reithmaier, J. P., Klopf, F., Schäfer, F. Fine structure of neutral and charged excitons in self-assembled In(Ga)As/(Al)GaAs quantum dots. *Phys. Rev. B* **65**, 195315 (2002).

[2] Yugova, I. A., Greilich, A., Zhukov, E. A., Yakovlev, D. R., Bayer, M., Reuter, D., Wieck, A. D. Exciton fine structure in InGaAs/GaAs quantum dots revisited by pump-probe Faraday rotation. *Phys. Rev. B* **75**, 195325 (2007).

[3] Schwan, A., Meiners, B.-M., Greilich, A., Yakovlev, D. R., Bayer, M., Maia, A. D. B., Quivy, A. A., Henriques, A. B. Anisotropy of electron and hole g-factors in (In,Ga)As quantum dots. *Appl. Phys. Lett.* **99**, 221914 (2011).

[4] Crooker, S. A., Brandt, J., Sandfort, C., Greilich, A., Yakovlev, D. R., Reuter, D., Wieck, A. D., Bayer, M. Spin Noise of Electrons and Holes in Self-Assembled Quantum Dots. *Phys. Rev. Lett.* **104**, 036601 (2010).

[5] Debus, J., Sapega, V. F., Dunker, D., Yakovlev, D. R., Reuter, D., Wieck, A. D., Bayer, M. Spin-flip Raman scattering of the resident electron in singly charged (In,Ga)As/GaAs quantum dot ensembles. *Phys. Rev. B* **90**, 235404 (2014).

[6] Debus, J., Kudlacik, D., Sapega, V. F., Dunker, D., Bohn, P., Paßmann, F., Braukmann, D., Rautert, J., Yakovlev, D. R., Reuter, D., Wieck, A. D., Bayer, M. Nuclear spin polarization in the electron spin-flip Raman scattering of singly charged (In,Ga)As/GaAs quantum dots. *Phys. Rev. B* **92**, 195421 (2015).

[7] Debus, J., Kudlacik, D., Waldkirch, P., Sapega, V. F., Scholz, S., Ludwig, A., Wieck, A. D., Bayer, M. Efficiency enhancement of the coherent electron spin-flip Raman scattering through thermal phonons in (In,Ga)As/GaAs quantum dots. *Phys. Rev. B* **95**, 201303(R) (2017).

[8] Lock, J. A., Laven, P. Co-polarized and cross-polarized scattering of an off-axis focused Gaussian beam by a spherical particle. 1. Exact GLMT formalism. *J. Quant. Spectrosc. Radiat. Transf.* **221**, 260-272 (2018).

[9] L. Brillouin. The Scattering Cross Section of Spheres for Electromagnetic Waves. *J. Appl. Phys.* **20**, 1110 (1949).

[10] Brennan, K., Hess, K. High field transport in GaAs, InP and InAs. *Solid-State Electronics* **27**, 347-357 (1984).

[11] Kunets, Vas. P., Teodoro, M. D., Dorogan, V. G., Lytvyn, P. M., Tarasov, G. G., Sleezer, R., Ware, M. E., Mazur, Yu. I., Krasinski, J. S., Salamo, G. J. Interface roughness scattering in laterally coupled InGaAs quantum wires. *Appl. Phys. Lett.* **97**, 262103 (2010).

[12] Lüker, S., Kuhn, T., Reiter, D. E. Phonon impact on optical control schemes of quantum dots: Role of quantum dot geometry and symmetry. *Phys. Rev. B* **96**, 245306 (2017).

[13] Sugaya, T., Bird, J. P., Ferry, D. K., Shimizu, T., Jang, K. Y., Ogura, M., Sugiyama, Y., Yonei, K. Electron-phonon scattering in an etched InGaAs quantum wire. *Physica B: Condensed Matter* **314**, 99-103 (2002).

[14] Gerlich, D. Elastic Constants of Single-Crystal Indium Arsenide. *J. Appl. Phys.* **34**, 2915 (1963).

[15] Cottam, R. I., Saunders, G. A. The elastic constants of GaAs from 2 K to 320 K. *J. Phys. C: Solid State Phys.* **6**, 2105 (1973).

[16] Cardona, M., Merlin, R. *Light Scattering in Solids IX*; pp. 282-298 (Springer, 2007).

[17] Norris, A. N. An inequality for longitudinal and transverse wave attenuation coefficients. *J. Acoust. Soc. Am.* **141**, 475-479 (2017).

[18] Comas, F., Studart, N. Electron-phonon interaction in quantum-dot/quantum-well semiconductor heterostructures. *Phys. Rev. B* **69**, 235321 (2004).

[19] Werschler, F., Hinz, C., Froning, F., Gumbsheimer, P., Haase, J., Negele, C., de Roo, T., Mecking, S., Leitenstorfer, A., Seletskiy, D.V. Coupling of Excitons and Discrete Acoustic Phonons in Vibrationally Isolated Quantum Emitters. *Nano Lett.* **16**, 5861-5865 (2016).

[20] Montagna, M., Dusi, R. Raman scattering from small spherical particles. *Phys. Rev. B* **52**, 10080 (1995).

[21] Zhou, D., Lorke, A. Wave functions of elliptical quantum dots in a magnetic field. *Am. J. Phys.* **83**, 205 (2015).

[22] Zielinski, M. Including strain in atomistic tight-binding Hamiltonians: An application to self-assembled InAs/GaAs and InAs/InP quantum dots. *Phys. Rev. B* **86**, 115424 (2012).

[23] Bester, G., Zunger, A., Wu, X., Vanderbilt, D. Effects of linear and nonlinear piezoelectricity on the electronic properties of InAs/GaAs quantum dots. *Phys. Rev. B* **74**, 081305(R) (2006).

[24] Saviot, L., Murray, D. B. Longitudinal versus transverse spheroidal vibrational modes of an elastic sphere. *Phys. Rev. B* **72**, 205433 (2005).

[25] Toporski, J., Dieing, T., Hollricher, O. *Confocal Raman Microscopy*; ch. 21 by T. Wermelinger and R. Spolenak, pp. 514-517 (Springer, 2018).

[26] E. L. Ivchenko, I. G. Lang, and S. T. Pavlov, Resonant secondary radiation in polar semiconductors, *physica status solidi (b)* **85**, 81 (1978),

[27] I. Paradisanos, G. Wang, E. M. Alexeev, A. R. Cadore, X. Marie, A. C. Ferrari, M. M. Glazov, and B. Urbaszek, Efficient phonon cascades in WSe_2 monolayers, Nature Communications **12**, 538 (2021).

[28] E. L. Ivchenko, *Optical spectroscopy of semiconductor nanostructures* (Alpha Science, Harrow UK, 2005).

[29] N. V. Kozyrev, I. I. Kozlov, B. R. Namozov, L. E. Golub, M. M. Glazov, Y. G. Kusrayev, and T. Wojtowicz, Double resonant Raman scattering and photoluminescence mediated by heavy and light hole excitons in a (Cd,Mn)Te quantum well in external magnetic field, *Phys. Rev. B* **108**, 245306 (2023).

[30] U. Fano, Effects of configuration interaction on intensities and phase shifts, *Phys. Rev.* **124**, 1866 (1961).
